# ALTERED MERISTEM PROGRAM1 sustains cellular differentiation by limiting HD-ZIP III transcription factor gene expression

**DOI:** 10.1093/plphys/kiae300

**Published:** 2024-05-23

**Authors:** Saiqi Yang, Olena Poretska, Brigitte Poppenberger, Tobias Sieberer

**Affiliations:** Research Unit Plant Growth Regulation, TUM School of Life Sciences, Technical University of Munich, DE-85354 Freising, Germany; Research Unit Plant Growth Regulation, TUM School of Life Sciences, Technical University of Munich, DE-85354 Freising, Germany; Professorship Biotechnology of Horticultural Crops, TUM School of Life Sciences, Technical University of Munich, DE-85354 Freising, Germany; Research Unit Plant Growth Regulation, TUM School of Life Sciences, Technical University of Munich, DE-85354 Freising, Germany

## Abstract

Plants show remarkable developmental and regenerative plasticity through the sustained activity of stem cells in meristems. Under certain conditions, pluripotency can even be reestablished in cells that have already entered differentiation. Mutation of the putative carboxypeptidase ALTERED MERISTEM PROGRAM1 (AMP1) in Arabidopsis (*Arabidopsis thaliana*) causes a set of hypertrophic phenotypes, indicating a defect in the suppression of pluripotency. A role of AMP1 in the miRNA-mediated inhibition of translation has previously been reported; however, how this activity is related to its developmental functions is unclear. Here, we examined the functional interaction between AMP1 and the Class III homeodomain-leucine zipper (HD-ZIP III) transcription factors, which are miRNA-controlled determinants of shoot meristem specification. We found that the HD-ZIP III transcriptional output is enhanced in the *amp1* mutant and that plant lines with increased HD-ZIP III activity not only developed *amp1* mutant-like phenotypes but also showed a synergistic genetic interaction with the mutant. Conversely, the reduction of HD-ZIP III function suppressed the shoot hypertrophy defects of the *amp1* mutant. We further provide evidence that the expression domains of HD-ZIP III family members are expanded in the *amp1* mutant and that this misexpression occurs at the transcriptional level and does not involve the function of miRNA165/166. Finally, *amp1* mutant–specific phenotypes cannot be mimicked by a general inhibition of miRNA function in the AMP1 expression domain. These findings lead us to a model in which AMP1 restricts cellular pluripotency upstream of HD-ZIP III proteins, and this control appears to be not directly mediated by the canonical miRNA pathway.

## Teaser

A putative carboxypeptidase controls cellular pluripotency upstream of HD-ZIP III transcription factors by limiting tissue-specific transcription rather than mediating microRNA-dependent inhibition.

## Introduction

The spatiotemporal transition of cells from a pluripotent to a differentiated state is fundamental for the development of all multicellular organisms. This process is not only responsible for the establishment of the body plan and the formation of organs but also ensures continuous cell renewal and tissue repair. During embryogenesis of vascular plants, stem cell niches (SCNs) for the continuous development of shoot and root organs are established. These apical meristems enable plants to grow postembryonically in an environmentally responsive manner. Apical meristems are organized in distinct zones, which harbor cells with different functions. In the shoot apical meristem (SAM), pluripotent stem cells are located in the central zone (CZ), and their identity is specified by an underlying organizing center (OC). These stem cells produce nonstem cell progeny, which enters differentiation programs at the meristem periphery in the process of organ formation (Eshed Williams 2021).

Several key factors involved in the functional compartmentalization of the SAM have been identified in Arabidopsis (*Arabidopsis thaliana*). The size and position of the SAM SCN are constantly calibrated by cytokinin and miR170/171 gradients, which restrict the expression of WUSCHEL (WUS) and HAIRY MERISTEM transcription factors (TFs) to the OC (Gordon et al. 2009; Zhou et al. 2018). Movement of WUS to the CZ mediates stem cell identity and causes expression of the mobile peptide CLAVATA3 (Yadav et al. 2011; Daum et al. 2014), which diffuses back to the deeper cell layers, where it restricts the WUS expression domain by binding to a diverse battery of different leucine-rich repeat receptor-like protein kinase complexes (Wang and Jiao 2023). Increased auxin signaling in the peripheral zone (PZ) in combination with mutually exclusive expression of members of the Class III homeodomain-leucine zipper (HD-ZIP III) and KANADI TFs predefines the morphogenetic zone in which polar transport–mediated auxin gradients determine the positioning of lateral organs (Caggiano et al. 2017; Burian et al. 2022).

However, less is known about the molecular mechanisms that actively suppress pluripotency in cells of the PZ (Gaillochet and Lohmann 2015). Microsurgery and laser ablation experiments across various species have uncovered that stem cells located in the central domain of the SAM exert control over the pluripotency status of their offspring in the meristem periphery (Pilkington 1929; Sussex 1952; Loiseau 1959; Reinhardt et al. 2003; Yadav et al. 2010). The elimination of the CZ induced the rapid formation of a secondary SCN through the respecification of PZ cells. This observation indicates that the SCN in the SAM actively inhibits the stem cell identity of PZ cells. This lateral inhibition-like mechanism of pluripotency ensures the maintenance of a single active SCN while simultaneously providing a protective instrument to sustain meristem function in case of SCN damage. A similar control of pluripotency is observed in the female gametophyte of Arabidopsis, where the egg cell actively preserves the differentiation status of neighboring synergid cells (Tekleyohans et al. 2017). Another well-documented instance of this phenomenon is the preservation of the suspensor cell identity in the early embryo. Suspensor cells maintain a latent pluripotent status and can transform into functional secondary embryos upon the abortion of the initial embryo (Radoeva and Weijers 2014). Currently, it remains unclear whether the same molecular mechanism drives lateral inhibition of pluripotency in these diverse developmental contexts or if independent systems are at work.

The putative carboxypeptidase ALTERED MERISTEM PROGRAM1 (AMP1) appears to play a key role in the lateral inhibition of pluripotency. Arabidopsis *amp1* mutants exhibit enlarged SAMs and form ectopic SCNs in the meristem periphery while maintaining the primary stem cell population (Huang et al. 2015). Analysis of *amp1* embryo development also revealed a failure in maintaining suspensor cell identity, with mutant suspensors proliferating and forming secondary embryos (Vidaurre et al. 2007; Poretska et al. 2020). Furthermore, *amp1* synergid cells exhibit a tendency to be misspecified as functional supernumerary egg cells, allowing double egg cell fertilization and twin embryo formation at the expense of endosperm development (Kong et al. 2015). Notably, in the control of shoot stem cell and synergid identity, AMP1 appears to act independently of previously described cell fate regulators (Huang et al. 2015; Kong et al. 2015; Tekleyohans et al. 2017).

AMP1 belongs to the M28B family of zinc-dependent metalloproteases (Helliwell et al. 2001). The protein contains an N-terminal transmembrane domain and resides to the endoplasmic reticulum (Li et al. 2013; Huang et al. 2015). While its precise biochemical function is not resolved (Poretska et al. 2016), AMP1 has been shown to be required for the miRNA-mediated control of translation (Li et al. 2013). Combined elimination of AMP1 with either RNA-DEPENDENT RNASE6 or the AMP1 paralog LIKE-AMP1 (LAMP1) causes a widespread accumulation of miRNA target proteins without affecting miRNA-triggered mRNA slicing. AMP1/LAMP1-dependent translational repression occurs specifically at the rough endoplasmic reticulum, where AMP1 partially colocalizes with AGO1 (Li et al. 2013). However, to date, it is unclear if and how this activity is functionally connected to the well-documented developmental roles of the protein since AMP1 appears to act at least in some contexts in a miRNA-independent manner (Fouracre et al. 2020) and there is no obvious phenotypic overlap of *amp1* with miRNA pathway mutants (Yang et al. 2021).

The low phenotypic similarity between *amp1* and miRNA pathway mutants might be caused by a locally restricted release of translational repression in the AMP1 expression domain, affecting only a subset of miRNA targets with a specific role in lateral inhibition of pluripotency. We could recently show that the stem cell hypertrophy in the *amp1* SAM is at least partially driven by ectopic expression of the AP2 TF RAP2.6L (Yang et al. 2018). RAP2.6L expression is under direct control of the miRNA-regulated family of HD-ZIP III TFs, whose protein levels have been shown to be upregulated in *amp1* (Li et al. 2013; Poretska et al. 2016; Yang et al. 2018).

Since HD-ZIP III proteins play a central role in shoot SCN specification and embryo patterning (Smith and Long 2010; Zhang, Lian, et al. 2017; Zhang, Tucker, et al. 2017), we asked to which extent AMP1 might exert its function in cell fate maintenance by specifically affecting this class of miRNA targets. Here, we show that AMP1 controls cellular pluripotency in an HD-ZIP III-dependent manner in different developmental contexts. Our data support a model in which AMP1 limits tissue-specific transcription of HD-ZIP III proteins rather than mediating miRNA 165/166-dependent inhibition of their translation. Finally, we also demonstrate that suspensor-specific expression of HD-ZIP III proteins triggers twin embryo formation in Arabidopsis at a high frequency.

## Results

### Expression of the HD-ZIP III direct target *LITTLE ZIPPER3* (*ZPR3*) is upregulated in *amp1*

**Figure 1.**
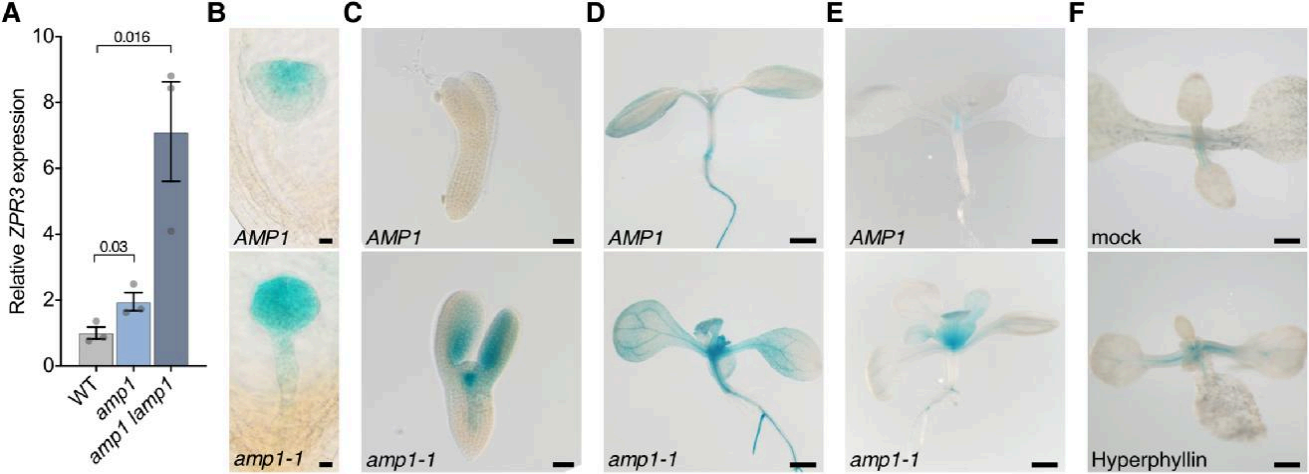
Expression of the HD-ZIP III direct target ZPR3 is upregulated in *amp1*. **A)** qPCR analysis of *ZPR3* expression in 8-d-old seedlings of the indicated genotypes (mean ± Se of the mean; *n* = 3). *P*-value is indicated above bars (unpaired Student's 2-tailed *t*-test). **B)** pZPR3::GUS activity in globular embryos of the indicated genotypes. **C)** pZPR3::ZPR3:GUS activity in torpedo-stage embryos of the indicated genotypes. **D)** pZPR3::GUS activity in 8-d-old seedlings of the indicated genotypes. **E)** pZPR3::ZPR3:GUS activity in 9-d-old seedlings of the indicated genotypes. **F)** pZPR3::ZPR3:GUS activity in 10-d-old WT Col-0 seedlings grown in liquid medium containing either 0.5% DMSO (mock) or 30 *μ*m hyperphyllin. Scale bars: 10 *μ*m **B)**, 50 *μ*m **C)**, and 1 mm **D**, **E**, and **F)**.

Based on overexpression analysis of reporter lines, it has been shown that HD-ZIP III proteins overaccumulate in *amp1* and *amp1 lamp1* in a miRNA-dependent manner (Li et al. 2013). To test whether this effect also results in a higher endogenous activity of these TFs, we monitored the expression level of the HD-ZIP III direct target *ZPR3* in *amp1*. In a previously performed transcriptomic analysis (Poretska et al. 2016), we found a 5-fold upregulation of *ZPR3* in *amp1-13*, and reverse transcription quantitative PCR (RT-qPCR) confirmed enhanced mRNA levels of *ZPR3* in *amp1-13* and *amp1 lamp1* (Fig. 1A). To resolve the changes in *ZPR3* expression at a tissue-specific level, we used the transcriptional GUS reporter *pZPR3::GUS* (Yang et al. 2022). Analysis of this reporter revealed that *ZPR3* transcription is already substantially expanded in *amp1-1* globular embryos (Fig. 1B). We also found an enhanced accumulation of ZPR3 protein in *amp1-1* embryos by monitoring the translational GUS reporter *pZPR3::ZPR3:GUS* (Fig. 1C; Wenkel et al. 2007). In *amp1-1* seedlings, *pZPR3::GUS* activity was stronger and broader in the shoot apex and vascular-associated tissues of the shoot (Fig. 1D). Similarly, the translational GUS reporter *pZPR3::ZPR3:GUS* showed a broadened and enhanced staining pattern in the shoot apex of *amp1-1* seedlings (Fig. 1E). The drug hyperphyllin provokes *amp1*-related morphological and molecular defects in wild-type (WT) seedlings (Poretska et al. 2016). Notably, hyperphyllin treatment also increased *pZPR3::ZPR3:GUS* activity in the shoot apex area and vascular bundles of petioles (Fig. 1F). Taken together, we found enhanced *ZPR3* expression, particularly in the shoot apex area of plants with compromised function of AMP1/LAMP1, which is already manifested during embryo development, indicating that the transcriptional activity of HD-ZIP III proteins is under control of these putative proteases.

### Plants with enhanced HD-ZIP III activity show phenotypic similarities to *amp1*

**Figure 2.**
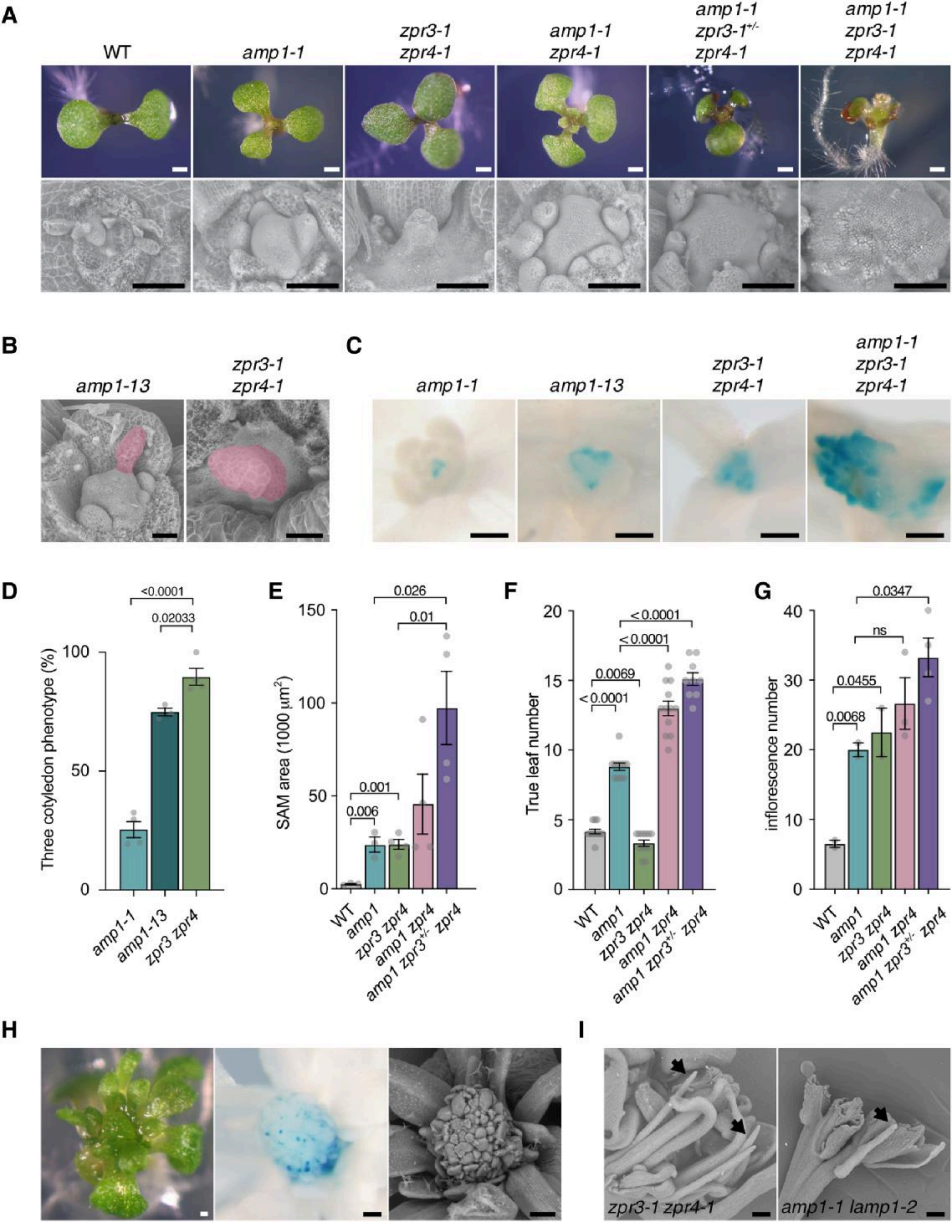
*Amp1* phenotypically resembles and genetically interacts with *zpr3 zpr4*. **A)** Seedling shoot phenotypes of indicated genotypes at 6 d after germination (DAG) (*upper panel*). Scanning electron micrographs of shoot meristems from 7-d-old seedlings of the indicated genotypes (*lower panel*). **B)** Scanning electron micrographs of shoot meristems from 6-d-old *amp1-13* and 12-d-old *zpr3-1 zpr4-1* seedlings showing radial outgrowths from the center of the SAM (labeled in red). **C)** Comparison of pWUS::GUS activities in 8-d-old seedlings of the indicated genotypes. **D)** Quantification of triple cotyledon formation rate in seedlings of the indicated genotypes (mean ± Se of the mean of 3 to 4 repeats; *n* ≥ 100). *P*-values are indicated above bars (unpaired Student's 2-tailed *t*-test). **E)** Meristem size quantification of 7-d-old seedlings (mean ± Se of the mean; *n* ≥ 3) *P*-values are indicated above bars (unpaired Student's 2-tailed *t*-test). **F)** Quantification of true leaf number in 7-d-old seedlings of the indicated genotypes (mean ± Se of the mean; *n* ≥ 9). *P*-values are indicated above bars (unpaired Student's 2-tailed *t*-test). **G)** Quantification of inflorescence number in 75-d-old plants of the indicated genotypes (mean ± Se of the mean; *n* ≥ 2). *P*-values are indicated above bars (unpaired Student's 2-tailed *t*-test). ns, not significant. **H)** Shoot meristem phenotype of a representative *amp1-1 zpr3-1 zpr4-1* plant at 12 DAG. Stereomicroscopic picture (*left*), pWUS::GUS activity (*middle*), and scanning electron micrograph (*right*). **I)** Scanning electron micrographs of flowers from the indicated genotypes. Radialized stamenoid organs are marked by arrowheads. Scale bars: 500 *µ*m (**A**, *upper panel*), 100 *µ*m (**A**, *lower panel*), 50 *μ*m **B)**, 250 *μ*m **C)**, and 200 *µ*m **H** and **I)**.

To further investigate the possible hyperactivity of HD-ZIP III proteins in *amp1* and its potential contribution to the developmental defects of the mutant, we assessed the level of phenotypic overlap between *amp1* and *zpr3-1 zpr4-1*. As direct suppressors of HD-ZIP III activity, the absence of these proteins results in a general derepression of HD-ZIP III function at the posttranslational level, a scenario also expected in *amp1*, based on its anticipated role in miRNA-dependent translation control (Wenkel et al. 2007; Kim et al. 2008; Li et al. 2013). *zpr3-1 zpr4-1* seedlings developed supernumerary cotyledons at an even higher frequency than the *amp1-13* null allele (Fig. 2, A and D). The vegetative SAM size of *zpr3-1 zpr4-1* was massively increased to a similar extent as in *amp1-1* (Fig. 2, A and E). Moreover, *zpr3-1 zpr4-1* seedlings formed a radialized organ-like bulge at a central apical position of the SAM (Fig. 2B; Kim et al. 2008), which is a hallmark of strong *amp1* alleles such as *amp1-13* (Fig. 2B; Poretska et al. 2020). This structure results from a ring-like formation of ectopic WUS-expressing stem cell pools (Fig. 2C), creating a central organogenic area able to produce radialized leaf-like protrusions.

The spectrum of overlapping developmental defects between *amp1* and *zpr3-1 zpr4-1* was not restricted to the vegetative growth phase. Also, the inflorescence number was significantly enhanced in *zpr3-1 zpr4-1* causing a comparable increase in bushiness as found in *amp1-1* (Fig. 2G). Since the *zpr3-1 zpr4-1* mutant, like *amp1-1 lamp1-2*, is fully sterile, we analyzed the flower morphology of both genotypes. Both lines showed drastic defects in flower organ development including the formation of pin-shaped stamens without anthers (Fig. 2I).

Taken together, we found a number of analogous shoot, inflorescence, and flower meristem alterations in *amp1-1* and *zpr3-1 zpr4-1* consistent with a model in which AMP1 controls shoot meristem organization by limiting HD-ZIP III overaccumulation and/or function.

### *Amp1* and *zpr3 zpr4* synergistically interact in the control of SAM integrity

In light of the phenotypic similarity between *amp1* and *zpr3 zpr4*, we proceeded to test their genetic interaction. First, we crossed the phenotypic *zpr4-1* single mutant with *amp1-1*. The resulting *amp1-1 zpr4-1* line showed a significantly increased true leaf number and also an elevated SAM size as compared with the *amp1-1* parental line (Fig. 2, A, E, and F). Mutating *ZPR3* in addition further enhanced these defects: the leaf number was nearly doubled in *amp1-1 zpr3-1^+/−^ zpr4-1* seedlings (Fig. 2, A and F), and their SAMs increased 4-fold in size in relation to *amp1-1* (Fig. 2E), whereas *zpr3-1^+/−^ zpr4-1* plants did not show any obvious alterations in these parameters. In the adult stage, a promoting effect of *zpr3-1^+/−^ zpr4-1* on the inflorescence number of *amp1* plants was also observed (Fig. 2G).

The triple homozygous mutant *amp1-1 zpr3-1 zpr4-1* developed a severely overproliferating shoot meristem with a completely distorted leaf initiation pattern, a phenotypic category neither seen in *amp1-1* nor in *zpr3-1 zpr4-1* (Fig. 2A). Consistent with the strong meristematic hypertrophy, pWUS::GUS activity was dramatically expanded in *amp1-1 zpr3-1 zpr4-1* and was present in multiple domains of the supersized SAM (Fig. 2C). At the stage of floral transition, triple mutant meristems showed several pWUS::GUS positive foci surrounded by small organ primordia (Fig. 2H), a WUS expression pattern that is highly reminiscent of *amp1-1 lamp1-2* meristems (Huang et al. 2015). Moreover, during embryo development, the elimination of ZPR3/ZPR4 function strongly increased the suspensor proliferation rate in *amp1-1* from 4% to 51%, which is even higher than that of the *amp1-1 lamp1*-2 double mutant (Supplementary Fig. S1, A and B).

### Limiting HD-ZIP III activity by ectopic expression of *ZPR3* suppresses *amp1* shoot phenotypes

**Figure 3.**
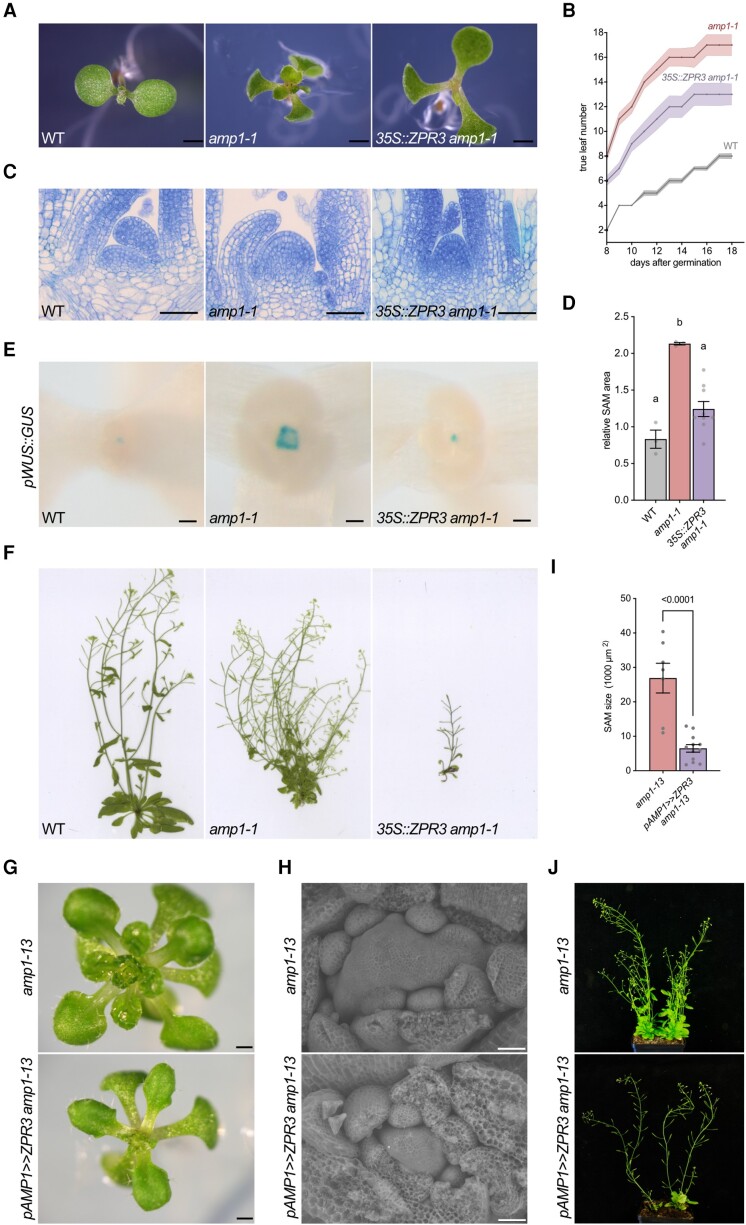
Limiting HD-ZIP III activity by ectopic *ZPR3* expression suppresses *amp1* shoot phenotypes. **A)** Seedling shoot phenotypes of indicated genotypes at 7 d after germination (DAG). **B)** Quantification of true leaf number in the indicated genotypes from 8 to 18 DAG (mean ± Se of the mean; *n* ≥ 13). **C)** Median longitudinal SAM sections from 7-d-old seedlings of the indicated genotypes. **D)** Quantification of SAM area from median longitudinal sections of 7-d-old seedlings of the indicated genotypes (mean ± Se of the mean; *n* ≥ 3). Different letters over the error bars indicate significant differences (*P* < 0.05; 1-way ANOVA followed by Tukey's multiple comparison tests). **E)** Comparison of pWUS::GUS activities in 5-d-old seedlings of the indicated genotypes. **F)** Shoot phenotypes of indicated genotypes at 35 DAG. **G)** Seedling shoot phenotypes of indicated genotypes at 10 DAG. **H)** Scanning electron micrographs of SAMs from 7-d-old seedlings of the indicated genotypes. **I)** SAM size quantification of 7-d-old seedlings (mean ± Se of the mean; *n* ≥ 3). The *P*-value is indicated above bars (unpaired Student's 2-tailed *t*-test). **J)** Shoot phenotypes of indicated genotypes at 50 DAG. Scale bars: 1 mm **A)**, 50 *µ*m **C** and **E)**, 500 *μ*m **G)**, and 50 *μ*m **H)**.

We next analyzed the impact of dampened HD-ZIP III activity on the shoot phenotype of *amp1* by crossing the mutant with *35S::ZPR3* (Wenkel et al. 2007). *ZPR3* overexpression mitigated the pronounced leaf formation rate of *amp1-1* (Fig. 3, A and B) and led to a normalization of SAM size, which was associated with a suppression of ectopic OC formation (Fig. 3, C to E). Moreover, the elevated branching phenotype of *amp1-1* was also completely abolished by *35S::ZPR3* (Fig. 3F). To assess, if ZPR3 overaccumulation, when restricted to the AMP1 expression domain, is also sufficient to weaken the SAM hypertrophy of the *amp1-13* null allele, we expressed ZPR3 under the control of the AMP1 promoter using a transactivation approach (Fig. 3G). *amp1-13* plants containing *pAMP1>>ZPR3* showed a clear rescue of the leaf initiation rate (Fig. 3G), the SAM overproliferation phenotype (Fig. 3, H and I), and the bushy shoot habitus at the adult stage (Fig. 3J), but did not exhibit the strong leaf polarity defects created by the ectopic overexpression of ZPR3 using the 35S::ZPR3 construct (Fig. 3, F, G, and J). Thus, repressing HD-ZIP III function in the *AMP1* expression domain alleviates *amp1*-associated SAM phenotypes at different developmental stages.

### Loss of REV/PHB/PHV function suppresses *amp1* shoot phenotypes

**Figure 4.**
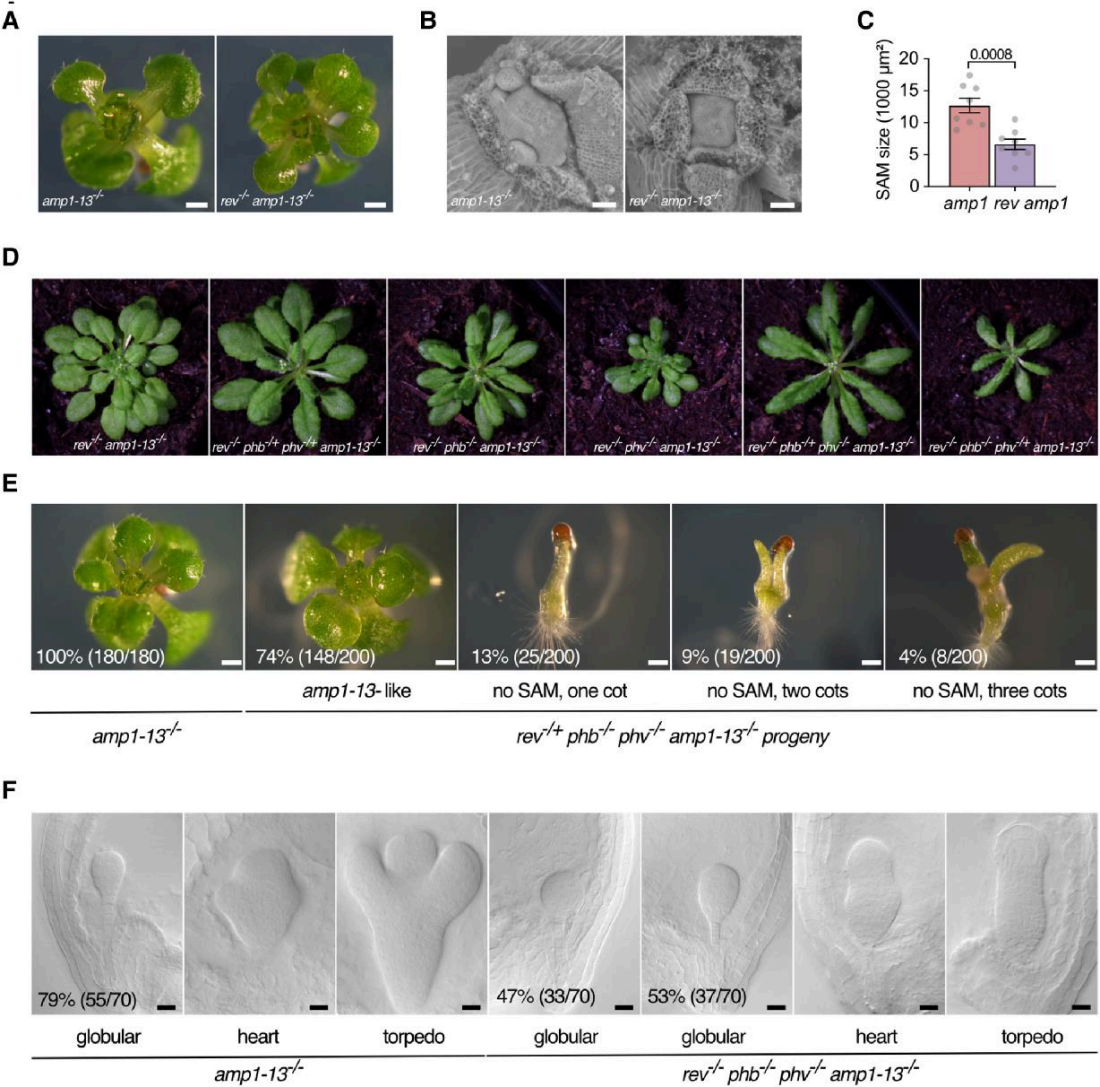
Loss of REV/PHB/PHV function suppresses *amp1* shoot phenotypes. **A)** Seedling shoot phenotypes of indicated genotypes at 10 d after germination (DAG). **B)** Scanning electron micrographs of SAMs from 4-d-old seedlings of the indicated genotypes. **C)** Quantification of SAM surface area from 4-d-old seedlings of the indicated genotypes (mean ± Se of the mean; *n* = 8). The *P*-value is indicated above bars (unpaired Student's 2-tailed *t*-test). **D)** Shoot phenotypes of the indicated genotypes at 20 DAG. **E)** Segregation analysis of shoot phenotypes in the 9-d-old selfing progeny of *amp1-13^−/−^* and *rev^−/+^ phb^−/−^ phv^−/−^ amp1-13^−/−^*. Frequency (%) and total number of seedlings showing the phenotype (in parentheses) are inserted. **F)** Embryo phenotypes of indicated genotypes. Frequency (%) and total number of seedlings showing the phenotype (in parentheses) are inserted. Scale bars: 500 *µ*m **A** and **E)**, 50 *µ*m **B)**, and 20 *μ*m **F)**.

To further assess to which extent HD-ZIP IIIs contribute to *amp1* phenotypes, we gradually eliminated REVOLUTA (REV), PHABULOSA (PHB), and PHAVOLUTA (PHV) in the *amp1-13* background. The *rev-6* mutation approximately halved the SAM surface area of *amp1-13* without noticeably affecting its leaf formation rate (Fig. 4, A to C). Stepwise introduction of *phb-13* and/or *phv-11* mutant alleles into the *rev-6 amp1-13* caused a noticeable further moderation of the *amp1* shoot phenotype (Fig. 4D). Finally, mutation of all 3 HD-ZIP III factors fully prevented SAM development in *amp1-13* (Fig. 4E;Supplementary Fig. S2). However, the formation of extra cotyledons and suspensor-to-embryo conversion was reduced but not fully eliminated in *rev-6 phb-13 phv-11 amp1-13* homozygous individuals (Fig. 4F;Supplementary Fig. S2).

### Suspensor-to-embryo conversion in *amp1* corresponds with ectopic activation of HD-ZIP III expression

**Figure 5.**
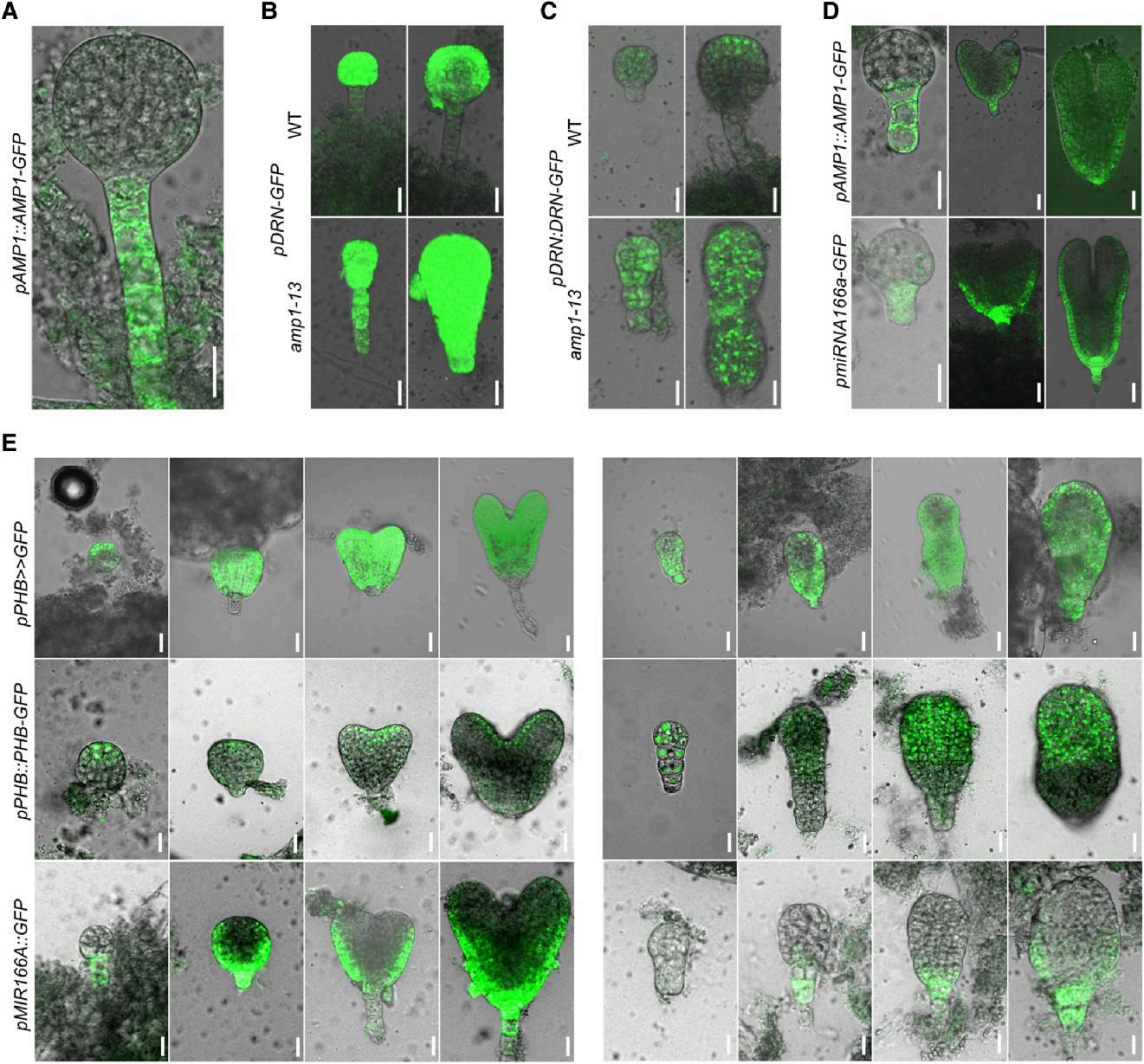
Suspensor-to-embryo conversion in *amp1* correlates with ectopic activation of HD-ZIP III expression. **A)** pAMP1::AMP1-GFP fluorescence in a WT embryo at the late globular stage. **B)** pDRN:GFP fluorescence in WT (*upper*) and *amp1-13* embryos (*bottom*) at the early globular (*left*) and late globular stage (*right*). **C)** pDRN:DRN-GFP fluorescence in WT (*upper*) and *amp1-13* embryos (*bottom*) at the early globular (*left*) and late globular stage (*right*). **D)** pAMP1::AMP1-GFP fluorescence (*upper*) and pmiRNA166a-GFP fluorescence (*bottom*) in globular (*left*), in heart stage (*middle*), and in torpedo stage embryos (*right*). **E)** Expression patterns of indicated GFP reporters at different developmental stages in WT (*left*) and *amp1-13* embryos (*right*). Scale bars: 20 *μ*m.

During embryogenesis, AMP1 is mainly expressed in the suspensor and later in the basal, peripheral areas of the embryo proper (Fig. 5A). In the absence of AMP1 function, suspensor cells start to proliferate, express the embryonic marker DORNRÖSCHEN (DRN) (Fig. 5, B and C), and are integrated in the development of the basal embryonic structures, whereas the original embryo proper contributes to an overdimensional SAM (Vidaurre et al. 2007; Poretska et al. 2020). Notably, the AMP1 expression pattern considerably overlaps with those of HD-ZIP III-specific miRNAs such as miRNA166a (Miyashima et al. 2013; Fig. 5D). To test whether AMP1 affects HD-ZIP III accumulation during embryogenesis, we compared PHB expression in WT and *amp1* embryos at different regulatory levels (Fig. 5E). The fluorescence of the transcriptional reporter pPHB>>GFP expanded to the proliferating suspensor cells of *amp1* embryos (Fig. 5E). This broader transcription domain coincided with a massive extension of the expression area of the translational reporters pPHB::PHB-GFP and gPHB::GUS, comprising the whole region of the original embryo domain (Fig. 5E; Supplementary Fig. S3). However, the basal embryonic area of *amp1*, in which pPHB>>GFP fluorescence was also present, did not show any signs of PHB::PHB-GFP accumulation. In this region, the expression of miRNA166A::GFP still took place in *amp1* but shifted more downward and appeared weaker compared with WT (Fig. 5E). Thus, suspensor-to-embryo conversion in *amp1* correlates with ectopic protein accumulation of PHB, which appears to result from a downward shift of PHB and miRNA166 transcription along the apical–basal axis, whereas the miRNA-mediated clearance of PHB accumulation seems to be still intact.

To further analyze the miRNA165/166-mediated control of HD-ZIP III expression in embryonic cotyledons of *amp1*, we monitored the distribution of the 35S::PHV-YFP reporter in the mutant. In both WT and *amp1-1*, the reporter only accumulated in the adaxial domains of the cotyledons of early and late torpedo-stage embryos (Supplementary Fig. S4, A and B). In *amp1*, the expression domain was slightly broader and slightly more intense; however, no reporter activity was visible in the abaxial domains where AMP1 is expressed.

### AMP1 restricts HD-ZIP III expression from the basis of the shoot meristem at least in part in a miRNA165/166-independent manner

**Figure 6.**
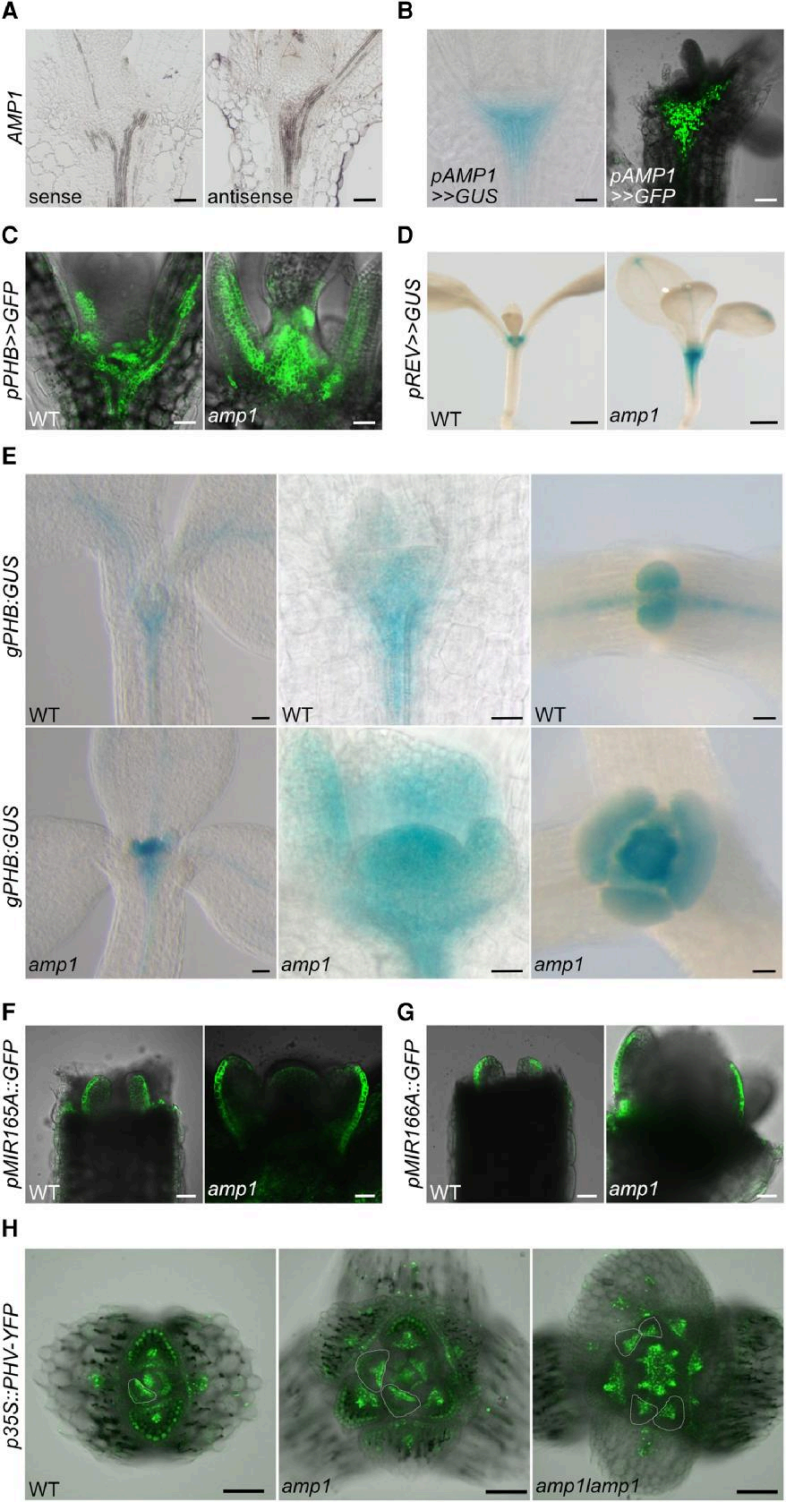
AMP1 restricts HD-ZIP III expression from the basis of the shoot meristem, which is not solely mediated by miRNA165/166. **A)** RNA in situ hybridization with median longitudinal shoot sections of 7-d-old WT seedlings using an AMP1-specific sense (*left*) and antisense probe (*right*). **B)** pAMP1>>GUS activity (*left*; 6 d after germination [DAG]) and pAMP1>>GFP fluorescence (*right*; 7 DAG) in shoots of WT seedlings. **C)** pPHB>>GFP fluorescence in shoots of 7-d-old WT and *amp1-13* seedlings. **D)** pREV::LhGR>>GUS activity in shoots of 8-d-old WT and *amp1-1* seedlings treated with 15 *μ*m dexamethasone for 20 h. **E)** gPHB::GUS activity in shoots of 4-d-old (*left* and *middle*) or 8-d-old (*right*) WT and *amp1-1* seedlings. **F)** pmiRNA165a-GFP fluorescence in SAMs of 4-d-old WT and *amp1-13* seedlings. **G)** pmiRNA166a-GFP fluorescence in SAMs of 4-d-old WT and *amp1-13* seedlings. **H)** p35S::PHV-YFP fluorescence in transversal shoot meristem sections of the indicated genotypes at 7 DAG. Dotted line indicates borders of leaf primordia. Scale bars: 50 *μ*m **A**, **B**, **C**, **E**, **F**, and **G)**, 500 *μ*m **D)**, and 100 *μ*m **H)**.

Next, we analyzed the impact of AMP1 on HD-ZIP III expression in the postembryonic shoot meristem. In 6-d-old seedling shoots, AMP1 expression was strongest in the vascular fork, which marks the boundary of the basal rib meristem (Fig. 6, A and B). Notably, in *amp1* mutant shoots, these areas showed enhanced expression of transcriptional reporters for PHB (Fig. 6C) as well as REV (Fig. 6D). Transcriptional upregulation of PHB was accompanied by prominent accumulation of the translational reporter gPHB:GUS in central and basal regions of the hypertrophic meristem of the mutant (Fig. 6E). However, the miRNA165/166-mediated clearance of PHB expression in the abaxial domains of leaf primordia appeared to be still functional in *amp1*. Moreover, in contrast to the situation in the embryo, we did not observe an obvious overlap between the expression domains of AMP1, miRNA165A, and miRNA166A in the seedling shoot meristem (Fig. 6, B, F, and G). Finally, expression of PHV-YFP under the control of the 35S promoter led to a relatively normal adaxial accumulation of the reporter protein in the leaf primordia of *amp1* and *amp1 lamp1*, but it more broadly accumulated in the enlarged SAM corpus of both mutants (Fig. 6H).

### HD-ZIP III overexpression under the control of the *AMP1* promoter provokes *amp1*-like phenotypes

**Figure 7.**
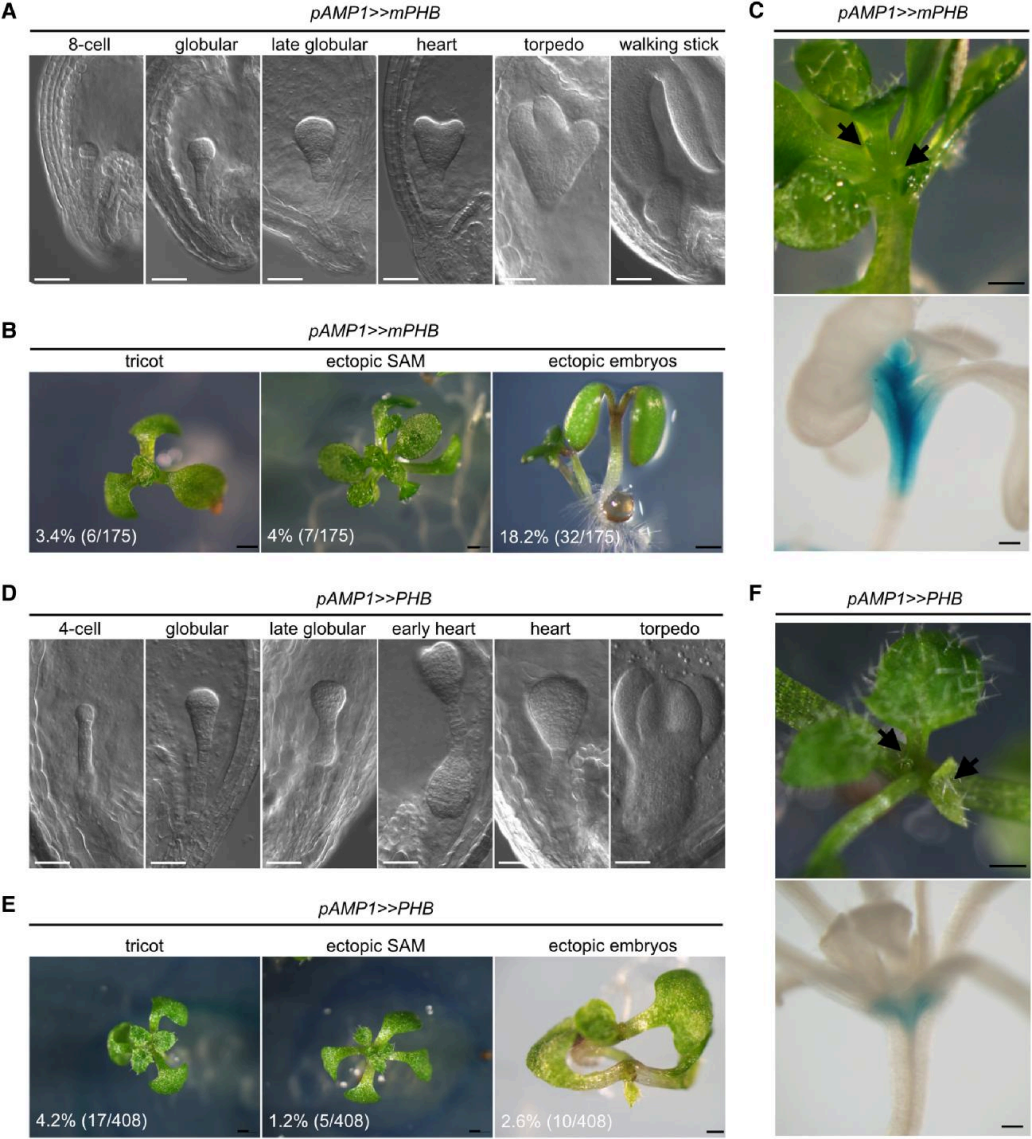
Misexpression of PHB under the control of the AMP1 promoter causes *amp1*-like phenotypes. **A)** Phenotypes of *pAMP>>mPHB* embryos at different developmental stages. **B)** Frequency of *amp1*-related phenotypes in *pAMP>>mPHB* seedlings at 8 d after germination (DAG). Percentages and total numbers of the indicated phenotypes are shown as insets. **C)***pAMP>>mPHB* seedling showing ectopic SAM formation (highlighted with arrows) at 9 DAG (*upper*). Analysis of GUS activity in the same seedling indicating expression domain of the pAMP1 driver (*lower*). **D)** Phenotypes of *pAMP>>PHB* embryos at different developmental stages. **E)** Frequency of *amp1*-related phenotypes in *pAMP>>mPHB* seedlings at 9 DAG. Percentages and total numbers of the indicated phenotypes are shown as insets. **F)***pAMP>>PHB* seedling showing ectopic SAM formation (highlighted with black arrows) at 9 DAG (*upper*). Analysis of GUS activity in the same seedling indicating expression domain of the *pAMP1* driver line (*lower*). Scale bars: 50 *μ*m **A** and **D)**, 500 *μ*m **B** and **E)**, 500 *μ*m (*upper* in **C** and **F**), and 200 *μ*m (*lower* in **C** and **F**).

To further assess whether the ectopic expression of HD-ZIP III proteins in *amp1* is causal for the pluripotency defects of the mutant, we expressed a miRNA-resistant version of PHB under the control of the *AMP1* promoter in the WT background. The direct fusion of the *AMP1* promoter with *mPHB-GFP* coding sequence (pAMP1::mPHB-YFP) did not result in obvious suspensor phenotypes but induced tricot formation (Supplementary Fig. S5, A and B). To achieve higher expression levels of the transgene, we also included a transactivation approach. *pAMP1>>mPHB* embryos showed fully penetrant suspensor proliferation associated with transgene expression (Supplementary Fig. S6), leading to *amp1*-like specification defects including axis elongation, hyperproliferation of the embryonic SAM, triple cotyledon formation, and twin embryo formation (Fig. 7A). As for *amp1*, these embryos developed into viable seedlings. Notably, 18% of seeds showed suspensor-derived twin seedling development, 3% of seedlings developed ectopic cotyledons, and 4% formed ectopic SAMs at an early seedling stage (Fig. 7, B and C).

To test whether these effects are miRNA-independent, we also expressed a miRNA165/166-sensitive WT version of PHB under the control of the *AMP1* promoter. In this line, we also observed suspensor proliferation that led to embryo axis elongation, triple cotyledon formation, and/or ectopic embryo formation (Fig. 7D). Postembryonically, triple cotyledon formation was at a comparable rate as in *pAMP>>mPHB*, whereas ectopic SAM formation and twin seedling formation dropped to 1.2% and 2.6%, respectively (Fig. 7, E and F). Taken together, overaccumulation of PHB in the *AMP1* expression domain triggers *amp1*-like pluripotency defects, and this is not strictly dependent on using a miRNA165/166-insensitive version of *PHB*.

### Compromising miRNA165/166 function in the *AMP1* expression domain does not create obvious *amp1*-like phenotypes

**Figure 8.**
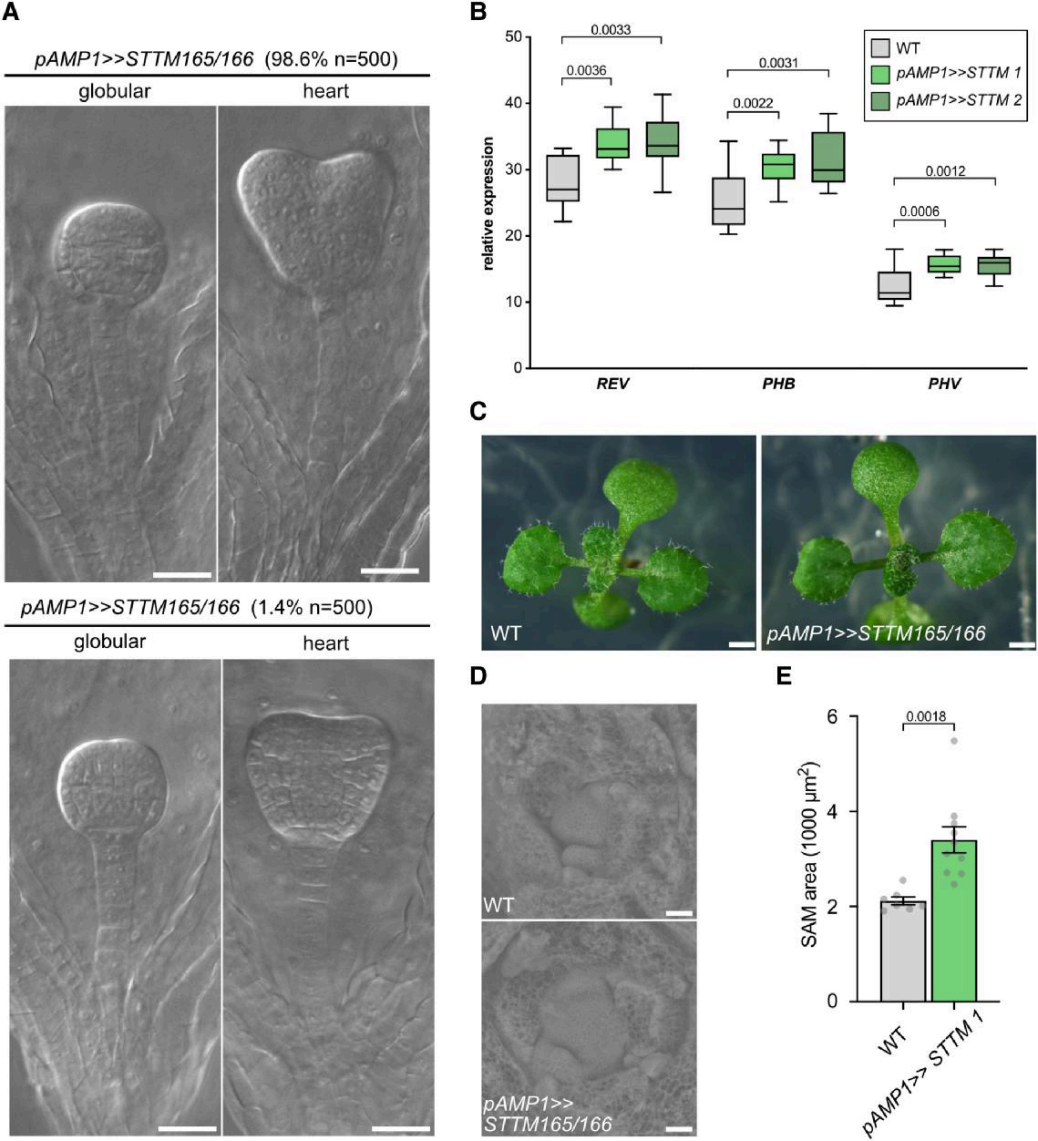
Compromising miRNA165/166 function in the *AMP1* expression domain does not create obvious *amp1*-like phenotypes. **A)** Photographs of *pAMP>>STTM165/166* globular and heart stage embryos showing WT phenotype (*upper*) or ectopic suspensor cell divisions (*lower*). Frequencies of shown phenotypes are indicated above the corresponding picture panels. **B)** qPCR analysis of *PHB*, *PHV*, and *REV* expression in 8-d-old seedlings of the indicated genotypes (center line, median; box limits, upper and lower quartiles; whiskers, minimal to maximal value; *n* = 12). *P*-values are indicated above bars (unpaired Student's 2-tailed *t*-test). **C)** Seedling phenotypes of the indicated genotypes at 12 d after germination. **D)** Scanning electron micrographs of shoot meristems from 13-d-old seedlings of the indicated genotypes. **E)** Meristem size quantification of 13-d-old seedlings (mean ± Se of the mean; *n* ≥ 7). The *P*-value is indicated above the error bar (unpaired Student's 2-tailed *t*-test). Scale bars: 20 *μ*m **A** and **D)** and 1 mm **C)**.

To further explore the role of miRNA165/166 in the instigation of *amp1* developmental defects, we blocked their function in the *AMP1* expression domain by combining the *pAMP1* driver line with a short tandem target mimic (STTM) effector line specific for miRNA165/166. Expression of the STTM165/166 construct leads to binding and subsequent degradation of miRNA165/166s (Yan et al. 2012). *pAMP1>>STTM165/166* embryos displayed only a marginal tendency for suspensor proliferation with 1.4% of embryos developing an extra tier of cells, which did not proceed in the formation of ectopic embryonic structures (Fig. 8A). The construct was functional since it led to a significant upregulation of *REV*, *PHB*, and *PHV* expression in the seedling stage (Fig. 8B) and caused leaf adaxialization phenotypes typical for defects in miRNA165/166 function (Fig. 8C). Although we detected a notable increase in SAM surface area, we did not observe the formation of twin seedlings, supernumerary cotyledons, or ectopic SAMs in *pAMP1>>STTM165/166* seedlings (Fig. 8, D and E).

### Compromising AGO1 function in the AMP1 expression domain does not provoke *amp1*-like phenotypes

**Figure 9.**
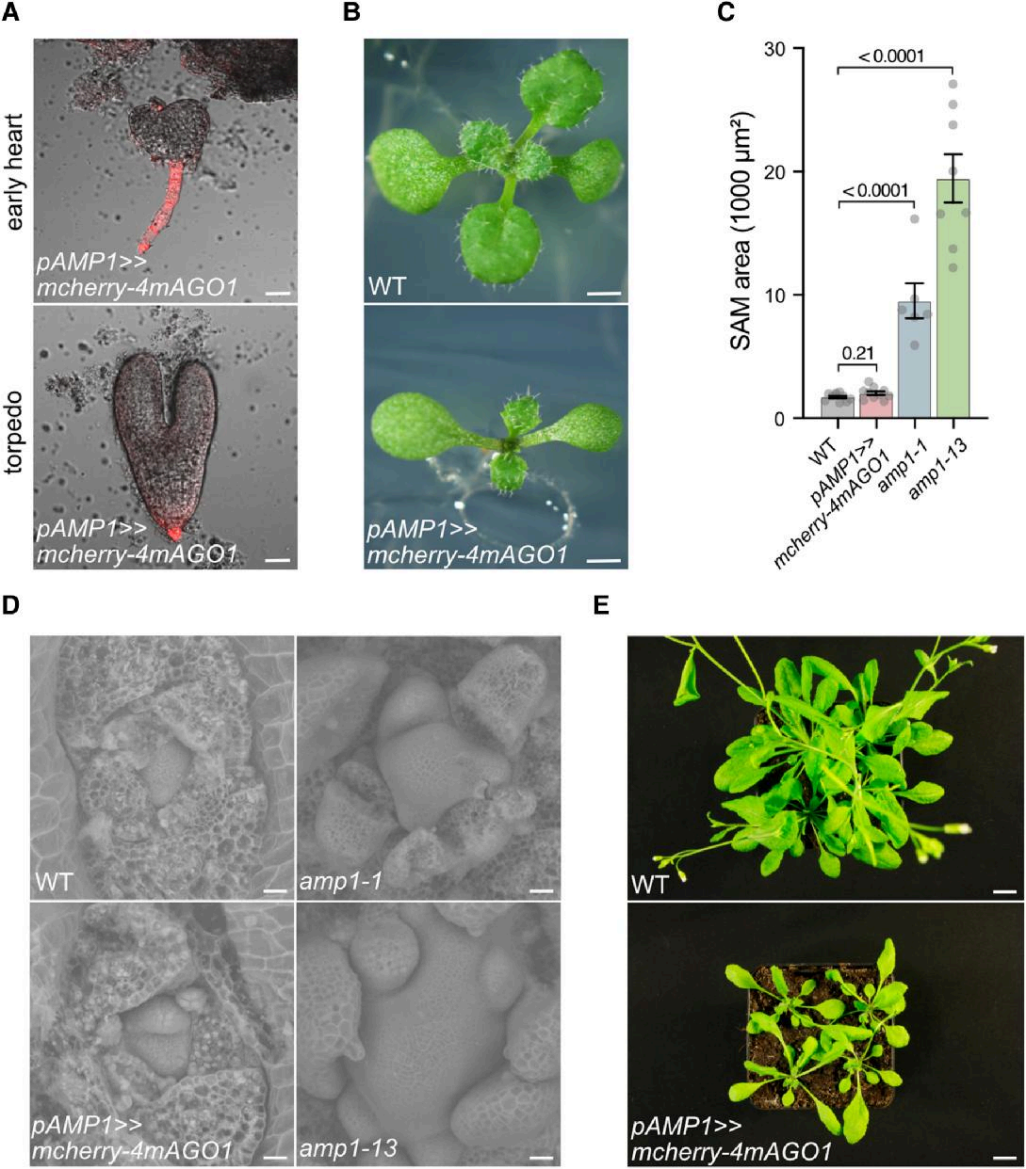
Compromising AGO1 function in the AMP1 expression domain does not provoke *amp1*-like phenotypes. **A)** The mCherry fluorescence in *pAMP1>>mcherry-4mAGO1* embryos of the indicated developmental stages. **B)** Seedling phenotypes of the indicated genotypes at 12 d after germination (DAG). **C)** Meristem size quantification of 8-d-old seedlings (mean ± Se of the mean; *n* ≥ 7). *P*-values are indicated above error bars (unpaired Student's 2-tailed *t*-test). **D)** Scanning electron micrographs of shoot meristems from 8-d-old seedlings of the indicated genotypes. **E)** Shoot phenotypes of WT and *pAMP1>>mcherry-4mAGO1* plants at 27 DAG. Scale bars: 20 *μ*m **A** and **D)**, 1 mm **B)**, and 1 cm **E)**.

Finally, to determine whether the *amp1* phenotype is mediated by a general release of miRNA target expression restricted to the AMP1 expression domain, we expressed a miRNA168-insensitive version of AGO1 (4mAGO1) under the control of the *AMP1* promoter. The resulting overaccumulation of AGO1 in the affected tissues is expected to cause a decrease in RNA-induced silencing complex (RISC) activity and subsequent developmental phenotypes specific to miRNA pathway mutants (Vaucheret et al. 2004; Deveson et al. 2013). We included an N-terminal mCherry tag in the expression cassette to prove the accumulation of the 4mAGO1 protein in the suspensor. Notably, strong expression of the transgene did not coincide with any obvious suspensor defects in our analysis (Fig. 9A). At the seedling stage, the transgene rather provoked *ago1*-like shoot phenotypes including increased leaf length/width ratio and a reduced leaf formation rate (Fig. 9, B and E) instead of mimicking any of the *amp1*-related shoot hypertrophic defects (Fig. 9, C and D).

### AMP1 shows only weak genetic interaction with core components of the miRNA pathway

To further investigate the functional relationship between AMP1 and the canonical miRNA pathway, we tested the genetic interaction of *amp1* with mutants in different core components of the miRNA machinery. At the seedling stage, none of the tested mutants in miRNA biogenesis (*se-3*, *dcl1-9*), miRNA transport (*hst-6*), or RISC function (*ago1-46*, *sqn-1*) significantly enhanced the development of twin seedlings, ectopic cotyledon formation, true leaf number, or rosette roundness in *amp1* but rather suppressed these phenotypes (Supplementary Fig. S7, A to E). Finally, mutation of *SUO1*, which specifically acts in the miRNA-dependent control of translation (Yang et al. 2012), did also not obviously reinforce the *amp1* mutant phenotype under the tested conditions (Supplementary Fig. S7, A to E).

## Discussion

The putative carboxypeptidase AMP1 has been identified as an important element of cell fate maintenance in different developmental contexts, such as synergid formation, embryo development, and postembryonic SAM patterning (Vidaurre et al. 2007; Huang et al. 2015; Kong et al. 2015). However, in contrast to the detailed knowledge about the *amp1* developmental aberrations, the precise biochemical activity of AMP1 is unknown, and there is only a fragmentary picture of the downstream components that mediate the enzyme's impact on cellular pluripotency. The revelation of AMP1's involvement in miRNA-related translation inhibition (Li et al. 2013) led to the speculation that the developmental defects of AMP1 are caused by misexpression of one or more miRNA targets in tissues where AMP1 is active. In this study, we tested the role of HD-ZIP III proteins as mediators of AMP1 function. We found that AMP1 suppresses embryonic identity in suspensor cells and stem cell identity in the shoot meristem periphery in an HD-ZIP III-dependent manner. However, our data support a mechanism limiting the transcription of HD-ZIP III proteins rather than directly acting through a miRNA165/166-related activity.

The finding that the AMP1-dependent restriction of shoot identity is mediated via the limitation of HD-ZIP III activity is supported by the previously described functions of this TF class in development. They have been shown to determine apical identity during early embryo patterning (Smith and Long 2010) and control the formation of the embryonic shoot SCN between the cotyledon primordia (Zhang, Lian, et al. 2017; Zhang, Tucker, et al. 2017). Moreover, a postembryonic increase of HD-ZIP III activity has been reported to cause shoot meristem hypertrophy phenotypes reminiscent of *amp1*, including increased SAM size, ectopic OC formation, and altered leaf initiation rate (McConnell et al. 2001; Williams et al. 2005; Wenkel et al. 2007; Kim et al. 2008). Later in development, REV is required for the formation of axillary shoot meristems (Talbert et al. 1995), a process also affected in *amp1*. Moreover, HD-ZIP III proteins are also rate-limiting factors for de novo shoot regeneration from root tissues (Zhang, Lian, et al. 2017; Zhang, Tucker, et al. 2017), a response that is also highly enhanced in *amp1* mutant plants (Yang et al. 2018). A functional connection between AMP1 and HD-ZIP III TFs is further supported by the reported REV-mediated suppression of AMP1 expression, which indicates the presence of a negative feedback loop between these factors controlling differentiation in the shoot meristem (Reinhart et al. 2013).

In addition, our work highlights a previously unknown role of HD-ZIP III proteins in the early separation of embryo and suspensor cell fates, which might be at least partially responsible for the suspensor-to-embryo conversion and twin embryo formation frequently detected in *amp1* mutants. This is consistent with the observation that several members of the miRNA165/166 family show strong suspensor-specific expression (Miyashima et al. 2013), largely overlapping with the expression pattern of AMP1 at this developmental stage, indicating that both factors might contribute to preventing HD-ZIP III accumulation in these tissues. When we expressed mPHB in the suspensor using the AMP1 promoter, it triggered twin seedling formation in 18% of seeds, outperforming the reported effect of suspensor-specific RKD1 expression in primary embryos or the effect of the *twn1* mutation (Radoeva et al. 2020). Thus, PHB represents a promising tool for plant biotechnological approaches in which the induction of embryonic identity is desired. Since AMP1 furthermore determines synergid specification in Arabidopsis (Kong et al. 2015), the question arises as to whether the putative protease also acts in this process in an HD-ZIP III-dependent manner.

Due to the reported role of AMP1 in miRNA-mediated translation repression, we expected that the increased activity of HD-ZIP III proteins in *amp1* results from reduced miRNA165/166 function. However, we found no solid evidence that this is the primary mechanism responsible for ectopic HD-ZIP III activity in the mutant. In the *amp1* embryo, the basal expansion of PHB protein accumulation is clearly associated with a broader expression of the corresponding transcriptional reporter and a simultaneous downward shift of the analyzed miRNA166a reporter. These observations rather support a model in which the absence of AMP1 leads to a basal relocation of the embryo/suspensor boundary by a process that acts upstream of miRNA165/166 function. Moreover, our suspensor-specific misexpression experiments of PHB revealed that suspensor-to-embryo conversion is not strictly dependent on using miRNA165/166-resistant versions of *PHB*. In addition, inhibition of miRNA165/166 function in the AMP1 expression domain using the STTM165/166 construct could not recapitulate this phenotype. A similar situation was also observed in the postembryonic shoot meristem, where miRNA165/166-dependent abaxial elimination of HD-ZIP III expression in leaf primordia appeared not to be compromised, whereas stronger transcription of REV, PHV, and PHB was most obvious in the CZ and the basal rib zone of the meristem, where we did not detect significant expression of the tested miRNA165/166 reporters.

One plausible explanation for this apparent miRNA165/166 independence of AMP1 function could be that AMP1 rather controls the translation of yet unidentified upstream miRNA targets, affecting the proper spatiotemporal expression of HD-ZIP III and/or miRNA165/166 genes during embryo and shoot meristem development. Numerous TFs have been previously identified as potential direct regulators of HD-ZIP III transcription by a yeast 1-hybrid approach (Gaudinier et al. 2011; Smit et al. 2020). Although most of these interactions have yet to be confirmed in planta, this group notably also includes targets of miRNA156/157, miRNA164, miRNA393, and miRNA396. However, the local inhibition of miRNA function in the AMP1 expression domain by a miRNA-resistant AGO1 version did not phenocopy any *amp1*-related developmental defects, which rather argues against a miRNA-dependent control of upstream TFs by AMP1. Nevertheless, we cannot exclude that this approach did not work due to the attributed specific role of AMP1 in miRNA-mediated inhibition of translation without affecting mRNA cleavage of miRNA targets (Li et al. 2013). With the availability of tissue-specific CRISPR applications (Decaestecker et al. 2019), it will be possible to test the phenotypic effects that arise from AMP1 promoter–driven mutation of other factors that also act specifically in the miRNA-specific inhibition of translation, like SUO1, DRB2, or KAT (Brodersen et al. 2008; Yang et al. 2012; Reis et al. 2015), to resolve this issue.

Alternatively, one could speculate that AMP1 controls the activity of upstream components of HD-ZIP III transcription, which are not under miRNA control. During embryogenesis, WOX TFs not only play a central role in determining embryo and suspensor identities but also mediate the establishment of shoot identity by promoting HD-ZIP III transcription (Zhang, Lian, et al. 2017; Zhang, Tucker, et al. 2017). Moreover, members of this TF family are also crucial in the auxin-mediated induction of somatic embryogenesis in tissue culture (Wang et al. 2020). Additional interesting contenders represent a subset of AUXIN RESPONSE FACTORS and members of the RKD family, which have been implicated in suspensor differentiation as well as in the cell fate control of the shoot meristem during development and in tissue culture (Rademacher et al. 2012; Ckurshumova et al. 2014; Krogan et al. 2016; Radoeva et al. 2020). Thus, it will be worthwhile to test to what extent AMP1 affects HD-ZIP III expression in dependence on any of these candidate TFs.

Another potentially interesting aspect of future research is the high similarity of shoot meristem defects between *amp1* and *zpr3 zpr4* and their strong synergistic genetic interaction. ZPRs repress HD-ZIP III activity at the posttranslational level by forming hetero-complexes with them (Wenkel et al. 2007; Kim et al. 2008). As direct targets of HD-ZIP III transcription, they constitute a negative feedback loop to moderate HD-ZIP III-dependent readouts (Husbands et al. 2016; Weits et al. 2019). Since our work shows that AMP1 limits the transcription of HD-ZIP III members, the related phenotypes of *amp1* and *zpr3/4* mutants might simply be caused by a similar spatiotemporal enhancement of HD-ZIP III activity mediated by defects at different regulatory levels. Alternatively, based on the reported partially antagonistic function of HD-ZIP III members (Prigge et al. 2005) that appears to be caused by mutual control of their transcription levels (Williams et al. 2005; Taylor-Teeples et al. 2015) in combination with reported different in vivo affinities between ZPR/HD-ZIP III complexes (Gruber et al. 2021), it could be speculated that the absence of ZPR3/ZPR4 might cause ectopic transcription profiles of HD-ZIP III submembers also found in *amp1*. A further level of complexity is added by the presence of the MEKHLA domain at the C-terminus of HD-ZIP III TFs that is thought to bind unidentified small molecule ligands, which impact homo-/hetero-dimerization behavior and thus their transcriptional output (Magnani and Barton 2011). Although we do not have any evidence at this stage, we cannot exclude that AMP1 affects HD-ZIP III function at this regulatory level.

Mutation of AMP1 also causes a drastic shortening of the plastochron and a delayed vegetative phase change (Chaudhury et al. 1993; Conway and Poethig 1997; Telfer et al. 1997). These phenotypic defects cannot be directly deduced from a malfunction of HD-ZIP III accumulation since plant lines with altered HD-ZIP III activity do not show such phenotypes. AMP1 thus must also act through HD-ZIP III-independent pathways to control SAM development. miRNA156-regulated SPL TFs are master regulators of both processes, suggesting that AMP1 might affect SPL activity (Wang et al. 2008; Wu et al. 2009). However, the phenotypic alterations in *amp1* are more congruent with a situation in which the activity of *mir156* is increased (Wang et al. 2008) rather than decreased, and a recent study showed that AMP1 controls vegetative phase change in a miRNA156/SPL-independent manner (Fouracre et al. 2020). In addition, this study demonstrated that AMP1 does not affect the miRNA-dependent translational repression of SPL9 or MYB33 when expressed under their endogenous promoters. Together with our findings, these data strongly indicate that AMP1 also exerts miRNA-independent functions in the control of development, which have yet to be revealed.

## Materials and methods

### Plant material and growth conditions

Unless stated otherwise, Arabidopsis (*A. thaliana* L. Heynh.) seeds were plated and plants grown as previously described (Yang et al. 2018).

*amp1-1* (N8324), *amp1-13* (N522988), *pt* (N235), *lamp1-2* (N110755), *hst-6* (N24279; Bollman et al. 2003), *sqn-1* (N67854; Berardini et al. 2001), *se-3* (N583196; Grigg et al. 2005)*, ago1-46* (N67862; Smith et al. 2009), *suo-4* (N67885; Yang et al. 2012), and *dcl1-9* (N3828; Jacobsen et al. 1999) were ordered from the Nottingham Arabidopsis Stock Centre (http://www.arabidopsis.info/). Additional published plant lines used in this study are as follows: *rev-6* and *rev-6 phb-13 phv-11* (Prigge et al. 2005); *pCLV3::GUS* and *pWUS::GUS* (Gross-Hardt et al. 2002); *zpr3-1* and *zpr4-1* (Wenkel et al. 2007); *35S::PHV-YFP* (Poretska et al. 2016); *pZPR::ZPR3::GUS* and *35S::ZPR3* (Wenkel et al. 2007); *pZPR3::GUS* (Yang et al. 2022); *pPHB>>GFP*, *pMIR165A::GFP*, and *pMIR166A::GFP* (Carlsbecker et al. 2010); *gPHB::GUS* (Gillmor et al. 2010); *pAMP1::AMP1-GFP* (Vidaurre et al. 2007); *pDRN::GFP* and *pDRN:DRN-GFP* (Cole et al. 2009); *pAMP1::LhG4* (Huang et al. 2015); *pREV::GR-LhG4* (Schurholz et al. 2018); and *pOP::GUS* (Craft et al. 2005). The corresponding plant lines in the *amp1-1* or *amp1-13* background were created by crossing.

### Gene constructs

PCR was performed with proofreading thermostable polymerase (Thermo Fisher Scientific, Waltham, MA, USA), and all clones were confirmed by sequencing. The resulting constructs were brought into Arabidopsis by the *Agrobacterium*-mediated floral dip transformation (Clough and Bent 1998).

To generate *pAMP1::mPHB-YFP*, we first inserted the silent G202G mutation (Mallory et al. 2004) into vector pGWR8-35S::PHB-YFP (Yang et al. 2022) by site-directed mutagenesis (Thermo Fisher Scientific, Waltham, MA, USA) with primers PHB G202G fwd/PHB G202G rev. Using KpnI and EcoRV, the 35S promoter was then exchanged against the AMP1 promoter (1,443-bp sequence upstream of start codon amplified with KpnIpAMP1f and EcoRVpAMP1r).

To create *pOP::PHB*, the PHB coding sequence was amplified with primers SalImPHBf and KpnImPHBr using pGWR8-35S::PHB-YFP as a template and inserted into pH-TOP (Craft et al. 2005) with SalI and KpnI. In pH-TOP, the pOP6 operator array functions as an orientation-independent enhancer element and coordinates the expression of a GUS reporter construct and inserted PHB construct simultaneously. Likewise, for *pOP::mPHB*, the mPHB^G202G^ coding sequence was amplified with primers SalImPHBf and KpnImPHBr using pGWR8-35S::mPHB-YFP as a template and inserted into pH-TOP with SalI and KpnI. For *pOP::ZPR3*, the ZPR3 coding sequence was amplified from complementary DNA (cDNA) with SalI-ZPR3f and KpnI-ZPR3r and inserted into pH-TOP with SalI and KpnI.

*pOP::mcherry-4mAGO1* and *pOP::STTM165/6* were created using the GreenGate system (Lampropoulos et al. 2013; Schurholz et al. 2018), ordered from Addgene (https://www.addgene.org, kit no. 1000000036). For *pOP::mcherry-4mAGO1*, the mCherry-4mAGO1 was designed as previously reported (Vaucheret et al. 2004). In brief, AGO1 coding sequence was cloned with primer combination AGO1mCf and AGO1mCr and attached the “C” and “D” overhangs. The miR168 recognition site and the BsaI recognition site were silently mutated using the following primer combinations: 4mAGO1f and 4mAGO1r, mAGO1BsaIf, and mAGO1BsaIr, respectively. Then, the following modules were combined in pGGZ001: pOP6 (Module A, pSW180a/pGGA016), mCherry linker (Module B), 4mAGO1 (Module C), D-dummy (Module D), UBQ10 terminator (Module E), and pNOS::BastaR:tNOS (Module F). For *pOP::STTM165/6*, the STTM165/6 sequence (Yan et al. 2012) was synthesized (Thermo Fisher Scientific, Waltham, MA, USA) with B and E overhangs resulting in the following DNA module: AACAGGTCTCaAACAGGGGGATGAAGCTACCTGGTCCGAGTTGTTGTTGTTATGGTCTAATTTAAATATGGTCTAAAGAAGAAGAATGGGGAATGAAGCTACCTGGTCCGACTGCaGAGACCTGTT. Then, the following modules were combined in pGGZ001: pOP6 (Module A, pSW180a/pGGA016), STTM165/6 (Module B), UBQ10 terminator (Module E), and pNOS::BastaR:tNOS (Module F).

### GUS staining

GUS staining was performed as previously described (Yang et al. 2018). The seedlings, embryos, or ovules were stained at 37 °C for various periods of time depending on the reporter strength. After staining, the tissue was dehydrated with 70% *v/v* ethanol. When necessary, the stained seedlings were cleared with a clearing solution (chloral hydrate/water/glycerol, 8:3:1, *v/v*). Samples were analyzed using a microscope (SZX10 or BX-61, Olympus).

### Phenotypic analysis of embryos

Whole-mount preparations were done as described (Berleth and Jürgens 1993). In brief, ovules from siliques of appropriate stages were fixed for 1 to 4 h in ethanol/acetic acid (6:1) at room temperature. After washing in 100% ethanol and 70% *v/v* ethanol, embryos were mounted in a mixture of chloral hydrate/glycerol/water (8:1:2) and cleared for about 1 h at room temperature. Images were taken with an Olympus BX61 microscope with differential interference contrast (DIC) optics.

### Leaf number analysis

The sequential appearance of the leaves was recorded in all experiments by visual observation of plants using a SZX10 stereomicroscope (Olympus) equipped with a DP26 digital camera (Olympus). For 10-d-old seedlings, a leaf was considered as initiated when its primordium could be observed under the stereomicroscope with 2× magnification.

### Histology

For transversal sections of shoot apices, 7-d-old Arabidopsis seedlings were freshly embedded in 7% low-melting-point agarose (Roth, Germany) and transversally sliced at 200-*μ*m thickness with a VF-100 vibratome (Precisionary Instruments, USA). The slices were collected in phosphate-buffered saline (137 mm NaCl, 2.68 mm KCl, 10 mm Na_2_HPO_4_, 2 mm KH_2_PO_4_, pH 7.4), mounted on microscopic slides (Marienfeld, Germany), and analyzed within 4 h using a FV1000 (Olympus, Japan) confocal laser scanning microscope (CLSM). Preparation and analysis of longitudinal sections of SAMs were performed as previously described (Yang et al. 2018).

### Fluorescence microscopy

Images were generated using a TCS SP8 (Leica Microsystems, Wetzlar, Germany) or a FV1000 (Olympus) CLSM. GFP was excited using a 488-nm argon laser, and emission was detected at 500 to 535 nm. Yellow fluorescent protein (YFP) was excited using a 515-nm argon laser, and emission was detected at 525 to 555 nm. The mCherry fluorescence was excited using a 561-nm diode laser and emission detected at 584 to 653 nm. Laser intensities and gains were not changed between compared samples.

### RT-qPCR

Approximately 50 mg of Arabidopsis seedling material was collected, snap-frozen in liquid nitrogen, and homogenized with a Retsch mill (Verder Scientific, Haan, Germany). RNA extraction, cDNA synthesis, and qPCR were performed as previously described (Yang et al. 2018). Sequences of oligos used for qPCR are shown in Supplementary Table S1. Data were normalized to *AtUBC* (*AT5G25760*) and measured in at least 3 technical replicates.

### Scanning electron microscopy

Freshly collected seedlings or flowers were incubated in formaldehyde/acetic acid (FAA) fixative (50% ethanol, 10% acetic acid, and 5% formaldehyde) overnight at 4 °C, dehydrated through a graded ethanol series, and subsequently subjected to supercritical point drying using an EM CPD300 (Leica, Wetzlar, Germany). Explants were mounted on conductive adhesive tabs (PLANO, Wetzlar, Germany). Pictures were taken with a T-3000 scanning electron microscope (Hitachi, Tokyo, Japan).

### RNA in situ hybridization

To generate AMP1 antisense and sense probes, the respective ORFs were amplified from cDNA using corresponding oligos (AMP1probeF, GCTCTCTTATCTTATCCTACGCACA/AMP1probeR, CGAAGAGACAAAGGCAAAGATGG), subcloned into pGEM-T Easy, and used as a template for transcription from the T7 or SP6 promoters. Ten-day-old Arabidopsis seedlings were fixed with FAA. The following paraffin infiltration was performed with a Leica ASP200 S tissue processor (Leica Biosystems). Sample preparations and in situ hybridizations of 7-*μ*m sections were performed as described (Sieber et al. 2004). Images were taken using a BX61 microscope (Olympus) with DIC optics.

### Statistics

All statistical parameters of the performed experiments are shown in the figures or figure legends, including the number of samples (*n*), type of statistical tests, and methods used. Statistical significance is denoted by lowercase letters, stars, or the shown *P*-values. Statistical analysis was performed with PRISM 8 software (GraphPad, San Diego, CA, USA).

### Accession numbers

Sequence data from this article can be found in the GenBank/EMBL data libraries under accession numbers AT3G54720 (AMP1), AT5G19740 (LAMP1), AT3G52770 (ZPR3), At2g36307 (ZPR4), AT2G34710 (PHB), At1g30490 (PHV), AT5G60690 (REV), AT2G17950 (WUS), At1g12980 (DRN), AT1G01183 (MIR165A), AT2G46685 (MIR166A), At1g48410 (AGO1), and AT5G25760 (UBC).

## Supplementary Material

kiae300_Supplementary_Data

## Data Availability

The data, including plant material, supporting this study’s findings are available from the corresponding author upon request.

## Supplementary data

The following materials are available in the online version of this article.

**Supplementary Figure S1.** Mutation of *ZPR3* and *ZPR4* enhances embryo defects in *amp1*.

**Supplementary Figure S2.** Mutation of *rev phb phv* causes shoot meristem loss in *amp1-13*.

**Supplementary Figure S3.** Suspensor-to-embryo conversion in *amp1* correlates with ectopic gPHB::GUS activity.

**Supplementary Figure S4.** miRNA165/166-dependent adaxial restriction of PHV expression appears to be normal in *amp1* embryonic cotyledons.

**Supplementary Figure S5.** Misexpression of *PHB* under the control of the *AMP1* promoter causes tricot formation.

**Supplementary Figure S6.** Transactivation of *mPHB* under the control of the *AMP1* promoter triggers suspensor cell proliferation.

**Supplementary Figure S7.** Genetic interaction of *AMP1* with different components of the miRNA machinery.

**Supplementary Table S1.** List of oligos used in this study.

## Dive Curated Terms

The following phenotypic, genotypic, and functional terms are of significance to the work described in this paper:

DRB2 Gramene: AT2G28380
DRB2 Araport: AT2G28380

MYB33 Gramene: AT5G06100
MYB33 Araport: AT5G06100

SPL9 Gramene: At2g42200
SPL9 Araport: At2g42200

ZPR4 Gramene: AT2G36307
ZPR4 Araport: AT2G36307

ZPR3 Gramene: AT3G52770
ZPR3 Araport: AT3G52770

AMP1 Gramene: AT3G54720
AMP1 Araport: AT3G54720
