# Supplementary material for: ALTERED MERISTEM PROGRAM1 sustains cellular differentiation by limiting HD-ZIP III transcription factor gene expression
Source: Plant Physiol. 2024 May 23;196(1):291–308. doi: 10.1093/plphys/kiae300 (PMC11376390; doi:10.1093/plphys/kiae300)
Supplement: kiae300_Supplementary_Data [file kiae300_supplementary_data.pdf]

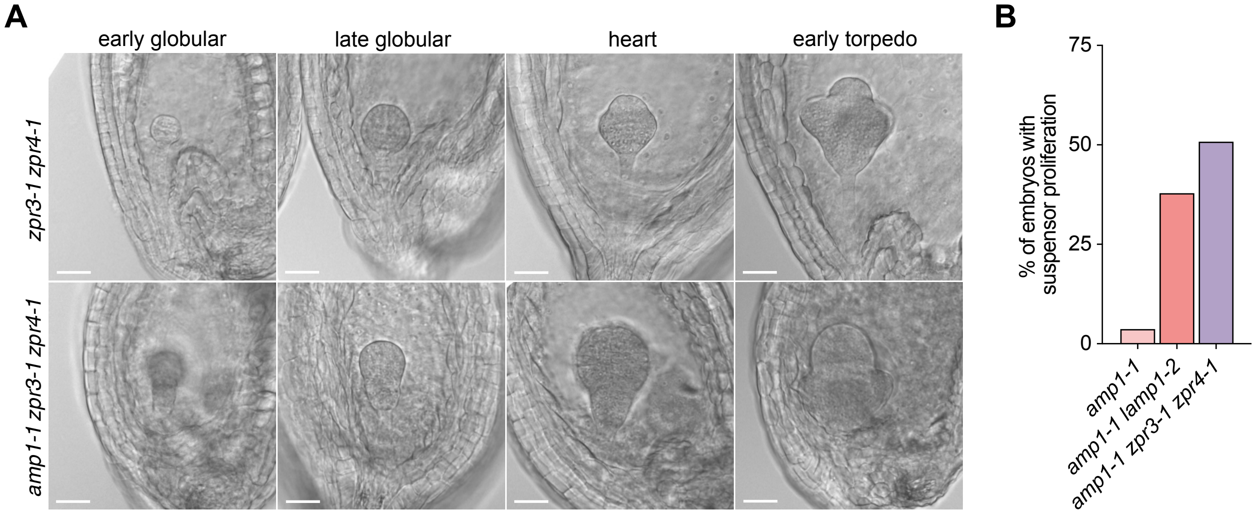

Supplementary Figure S1. Mutation of *ZPR3* and *ZPR4* enhances embryo defects in *amp1*. **(A)** *zpr3-1 zpr4-1* embryos and *amp1-1 zpr3-1 zpr4-1* embryos at the indicated developmental stages. **(B)** Frequency of suspensor proliferation phenotype in embryos of the indicated genotypes at the late globular stage. Scale bars: 50 µm.

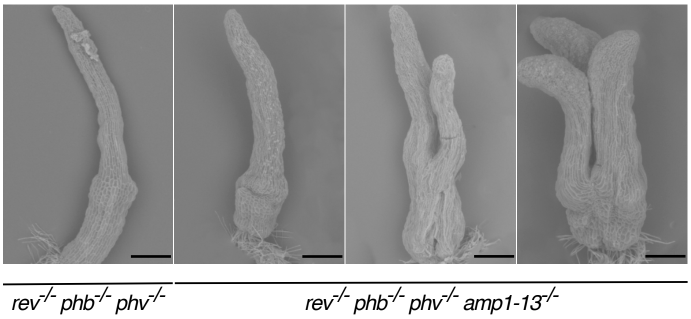

Supplementary Figure S2. Mutation of *rev phb phv* causes shoot meristem loss in *amp1-13*. Scanning electron micrographs of 12-d-old seedlings of the indicated genotypes. Scale bars: 500 μm.

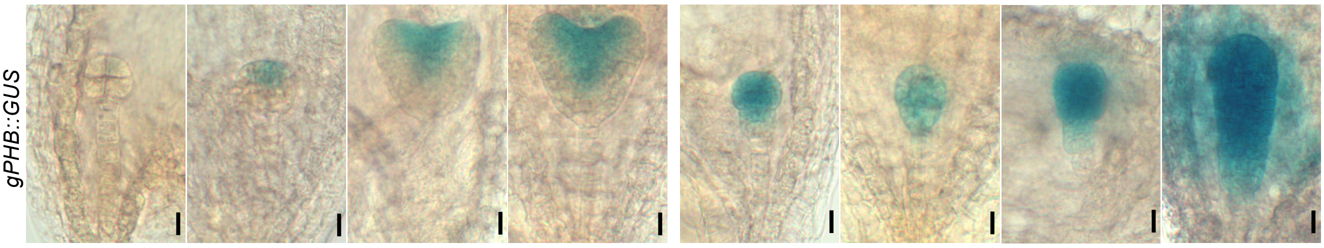

Supplementary Figure S3. Suspensor to embryo conversion in *amp1* correlates with ectopic gPHB::GUS activity. gPHB::GUS activity at different developmental stages in WT (left panel) and *amp1-13* embryos (right panel). Scale bars: 20 µm.

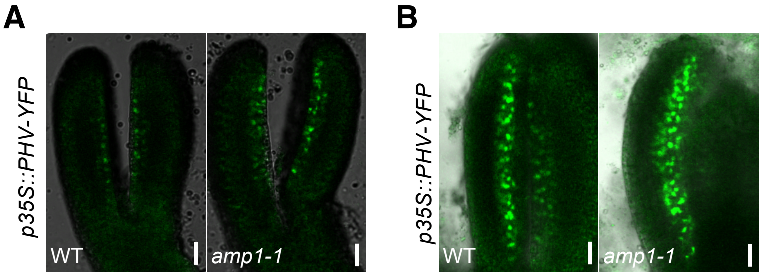

Supplementary Figure S4. miRNA165/166-dependent adaxial restriction of PHV expression appears to be normal in *amp1* embryonic cotyledons. **(A)** p35S::PHV-YFP fluorescence in wild-type coledylons (left) and *amp1-1* cotyledons (right panel) at the early torpedo stage. **(B)** p35S::PHV-YFP fluorescence in wild-type coledylons (left) and *amp1-1* cotyledons (right panel) at the late torpedo stage. Scale bars: 20 µm.

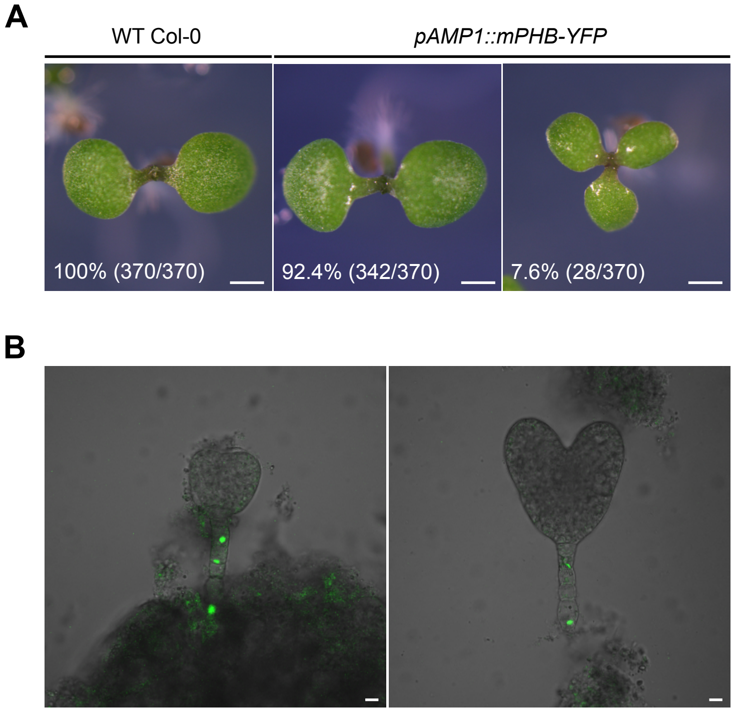

Supplementary Figure S5. Mis-expression of *PHB* under the control of the *AMP1* promoter causes tricot formation. **(A)** Frequency of tricot formation in *pAMP::mPHB-YFP* seedlings at 7 DAG. Percentages and total numbers of the indicated phenotypes are shown as inserts. **(B)** pAMP::mPHB-YFP fluorescence in late globular (left) and heart stage (right) embryos. Scale bars: 500 µm (A) and 10 µm (B).

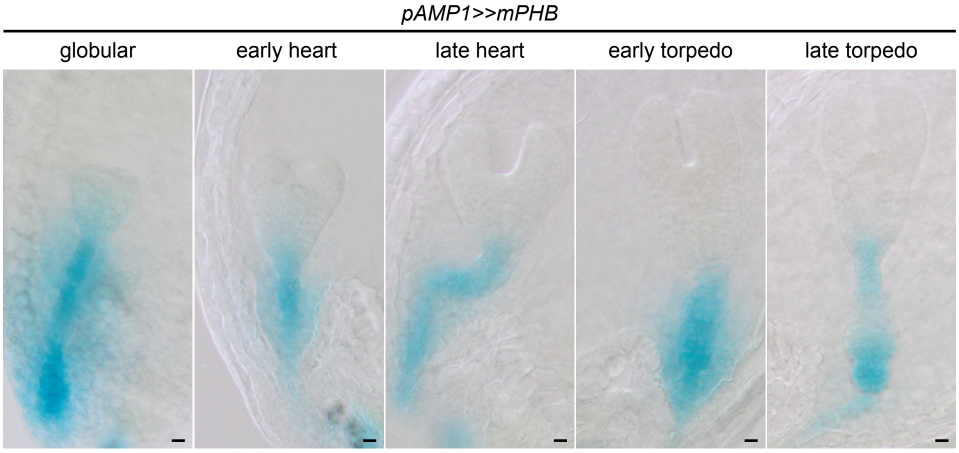

Supplementary Figure S6. Transactivation of *mPHB* under the control of the *AMP1* promoter triggers suspensor cell proliferation. Analysis of GUS activity in *pAMP>>mPHB* embryos at different developmental stages, indicating overlapp of the *pAMP1* driver line expression domain with suspensor cell proliferation. Scale bars: 50 μm.

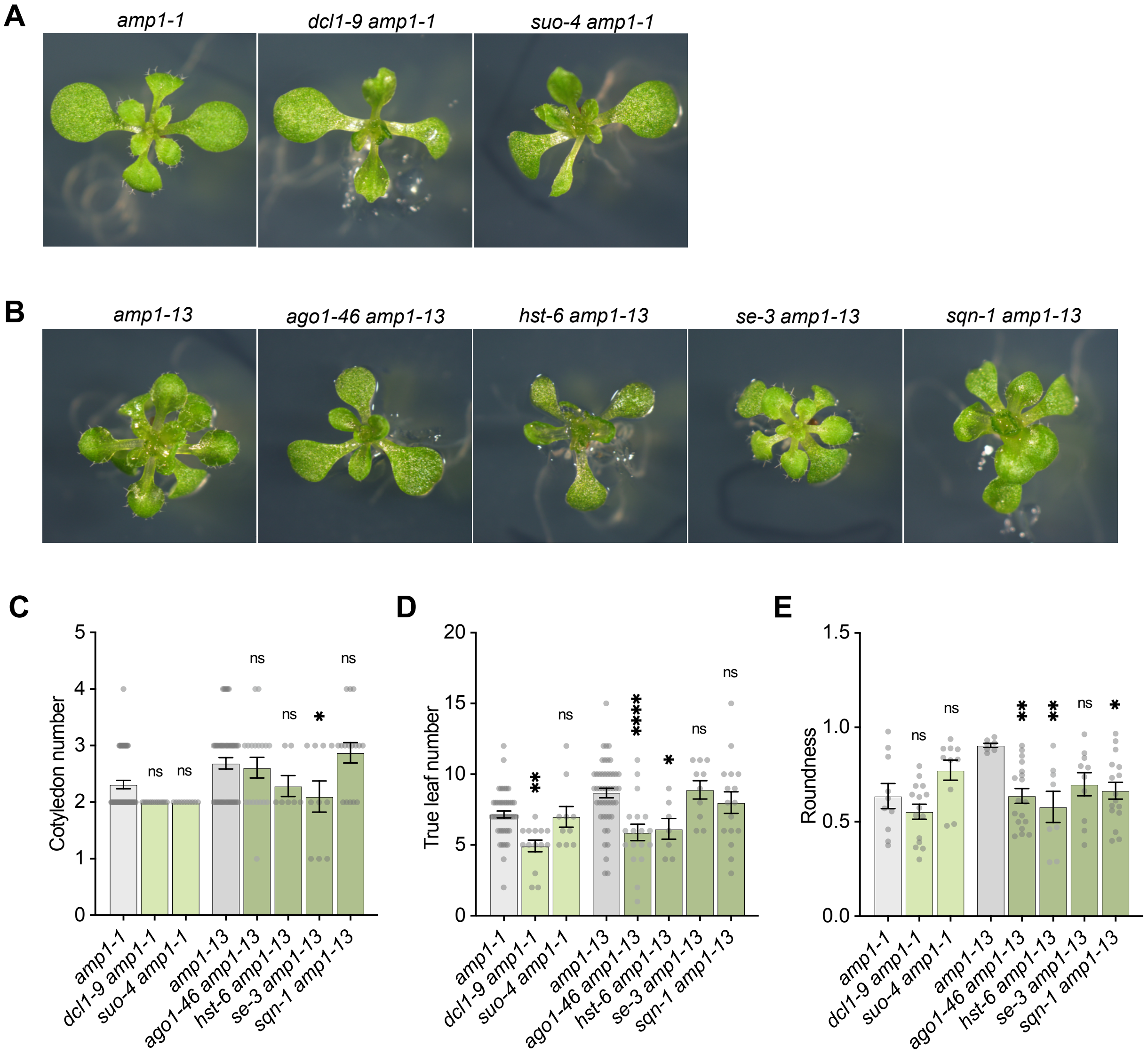

Supplementary Figure S7. Genetic interaction of *AMP1* with different components of the miRNA machinery. **(A)** Seedling shoot phenotypes of *amp1-1* and the indicated mutant combinations at 10 DAG. **(B)** Seedling shoot phenotypes of *amp1-13* and the indicated mutant combinations at 10 DAG. **(C)** Quantification of cotyledon number in seedlings of the indicated genotypes at 7 DAG (means ± SE of the mean; n ≥ 7); Asterisks represent statistical significance; ns, not significant; *, P< 0.05; ** (Unpaired Student's two-tailed t-test). **(D)** Quantification of true leaf number in seedlings of the indicated genotypes at 9 DAG (means ± SE of the mean; n ≥ 7); Asterisks represent statistical significance; ns, not significant; **, P < 0.005; ****, P < 0.0001; (Unpaired Student's two-tailed t-test). **(E)** Quantification of rosette roundness in seedlings of the indicated genotypes at 9 DAG (means ± SE of the mean; n ≥ 8) Asterisks represent statistical significance; ns, not significant; * P< 0.05; ** P < 0.005; (Unpaired Student's two-tailed t-test).

Supplementary Table S1. List of Oligos used in this study

Name	Sequence	Use
PHV G198G fwd	GGATGAAGCCTGGACCGGATTCTATTG	mutagenesis
PHV G198G rev	CAATCATCTGGACCCAGTCGACAGCAGT	
PHB G202G fwd	GGATGAAGCCTGGACCGGATTCTATTG	mutagenesis
PHB G202G rev	CAATCATCTGAACCCAGTCAACAGCAGT	
PHV mid F	GCAACTGCAGTGGAATAGCA	sequencing
PHV mid R	GTGGAACCATCTAGGGACGA	
PHB mid F	TTGGCATAGTCGCTATTTCG	sequencing
PHB mid R	AGCTTGACGTGTGGATCCTT	
KpnIpAMP1f	GGTACC AGAAGAAAGGAAGAGGAGGGAGA	cloning
EcoRVpAMP1r	GATATC TGCAGAGAGAGAGAGAGAGT	
SalImPHBf	GTCGAC ATGATGATGGTCCATTCGATGAG	cloning
KpnImPHBr	GGTACC TCAAACGAACGACCAATTCACG	
SalI-ZPR3f	GTCGAC ATGGAGAGGCTAAACTCGAAGC	cloning
KpnI-ZPR3r	GGTACC TCAAGATTGTCCAGAAGCAGAGC	
AGO1mCf	AACA GGTCTC A GGC TCA ACAATGGTGAGAAAGAGAAGAACGGA	cloning
AGO1mCr	A AACA GGTCTC T C TGA GCAGTAGAACATGACACGCT	
4mAGO1f	CTcCATCAgGCTACCTCACCTACTTAT	mutagenesis
4mAGO1r	CTCcGGcACTGATTGTCTCTGCGGTG	
mAGO1BsaIf	TGAAGGCTCTGGcTCTCGTGAAGCTGG	mutagenesis
mAGO1BsaIr	CCTCCTTCAGATGGAGCATCCGTTCTT	
AGO1midF	CTGGTCCACTTCCCTTTAACTCC	sequencing
AGO1midR	GCCACCAGTCACCACACC	
pOp6_F	TGCATATGTCGAGCTCAAGAA	sequencing
pOp6_R	CTTATATAGAGGAAGGGTCTT	
pGGZf	GGCCGCAACAGATCGTTTTA	sequencing
pGGZr	ATAGTCCTGTCGGGTTTCGC	
qZPR3f	TCTGCTTCTCAACACTCCTTCCC	qPCR
qZPR3r	CAACAAACAGCTTCGAGTTTAGCC	
qREVF	TCGATGAATCGGGTCGTAAGGC	qPCR
qREVR	CAAGCAAGCAAATCCCTGTTGC	
qPHBF	AAGACCCTTGACGAACCTGGTC	qPCR
qPHBR	GCAGATGAGCATAGCCCTGTTG	
qPHVF	ACTTGATGACTCTGGTCGTAGAGC	qPCR
qPHVR	CGGAAGATTCGCATATCCCTGCTG	
UBCF	TCAAATGGACCGCTCTTATC	qPCR
UBCR	CACAGACTGAAGCGTCCAAG	
